# Supplementary material for: Neurodevelopmental copy-number variants increase risk of internalizing and cardiometabolic multimorbidity: Findings from the UK Biobank
Source: Am J Hum Genet. 2026 Apr 6;113(6):1319–29. doi: 10.1016/j.ajhg.2026.02.021 (PMC13277688; doi:10.1016/j.ajhg.2026.02.021)
Supplement: Document S1. Figures S1–S6, Tables S1–S14, and supplemental notes [file mmc1.pdf]

The American Journal of Human Genetics, Volume *113*

Supplemental information

Neurodevelopmental copy-number variants increase risk of internalizing and cardiometabolic multimorbidity: Findings from the UK Biobank

Ioanna K. Katzourou, LINC consortium, Inês Barroso, Julie Clayton, Golam Khandaker, Daniel Stow, Nicolas Timpson, Ruby Tsang, Jack Underwood, Megan Wood, George Kirov, James Walters, Michael J. Owen, Peter Holmans, and Marianne B. M. van den Bree

SUPPLEMENTAL NOTES

The lists of clinical codes curated by the MULTIPLY project (https://zenodo.org/record/7643566) were used to establish instances of the conditions of interest(1). The primary care codelists for depression and anxiety were amended by psychiatrists within the LINC team. Dyslipidemia is not defined within the MULTIPLY clinical code lists, therefore the clinical code list from Baksh *et al*. was used to identify instances of dyslipidemia within primary care records(2), and ICD-10 codes E78.0, E78.1, E78.2, E78.3, E78.4, E78.5, E88.81 were used to define dyslipidemia in HES.

SUPPLEMENTAL FIGURES

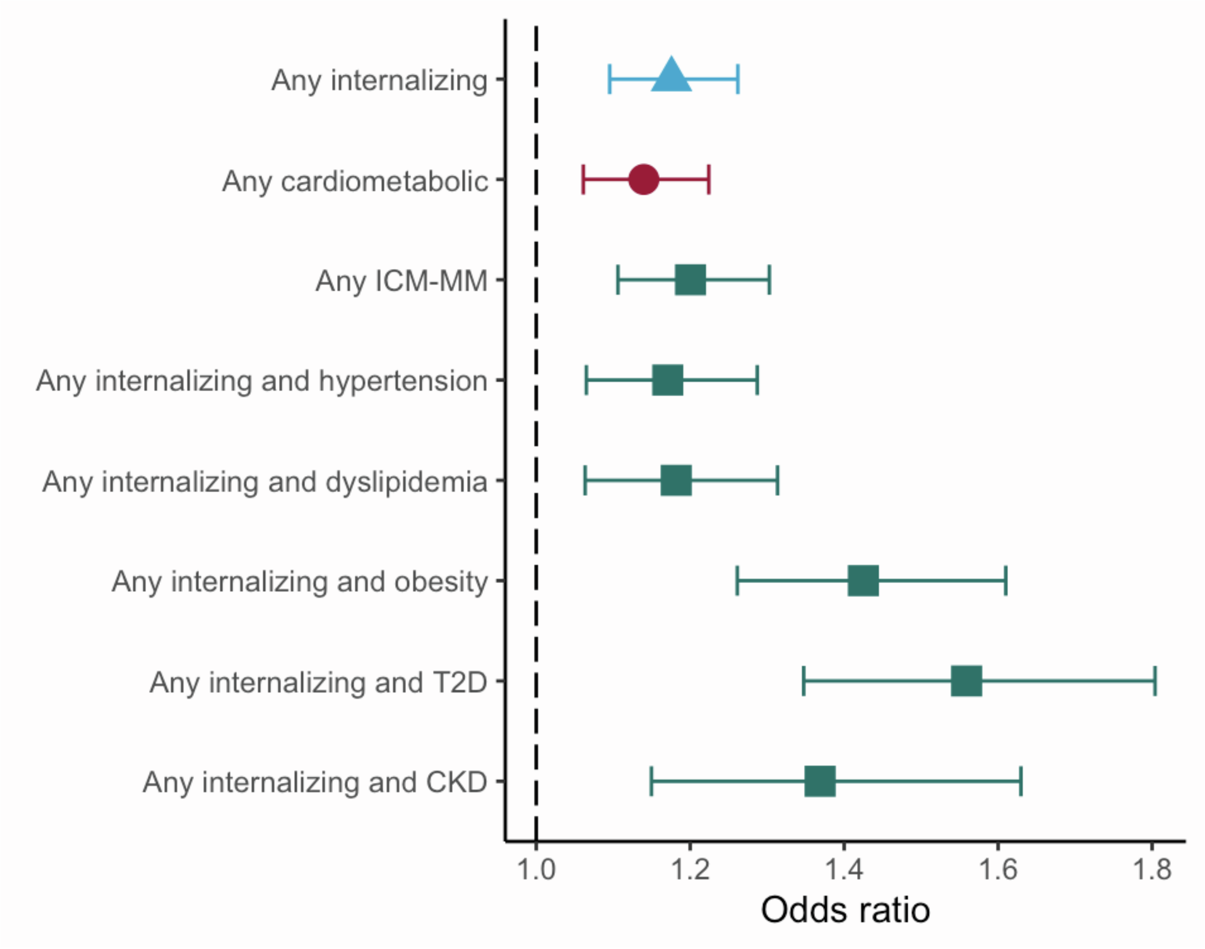

Figure S1. Association of ND-CNVs with multimorbidity for individuals with both primary care and HES data (N= 229,951).

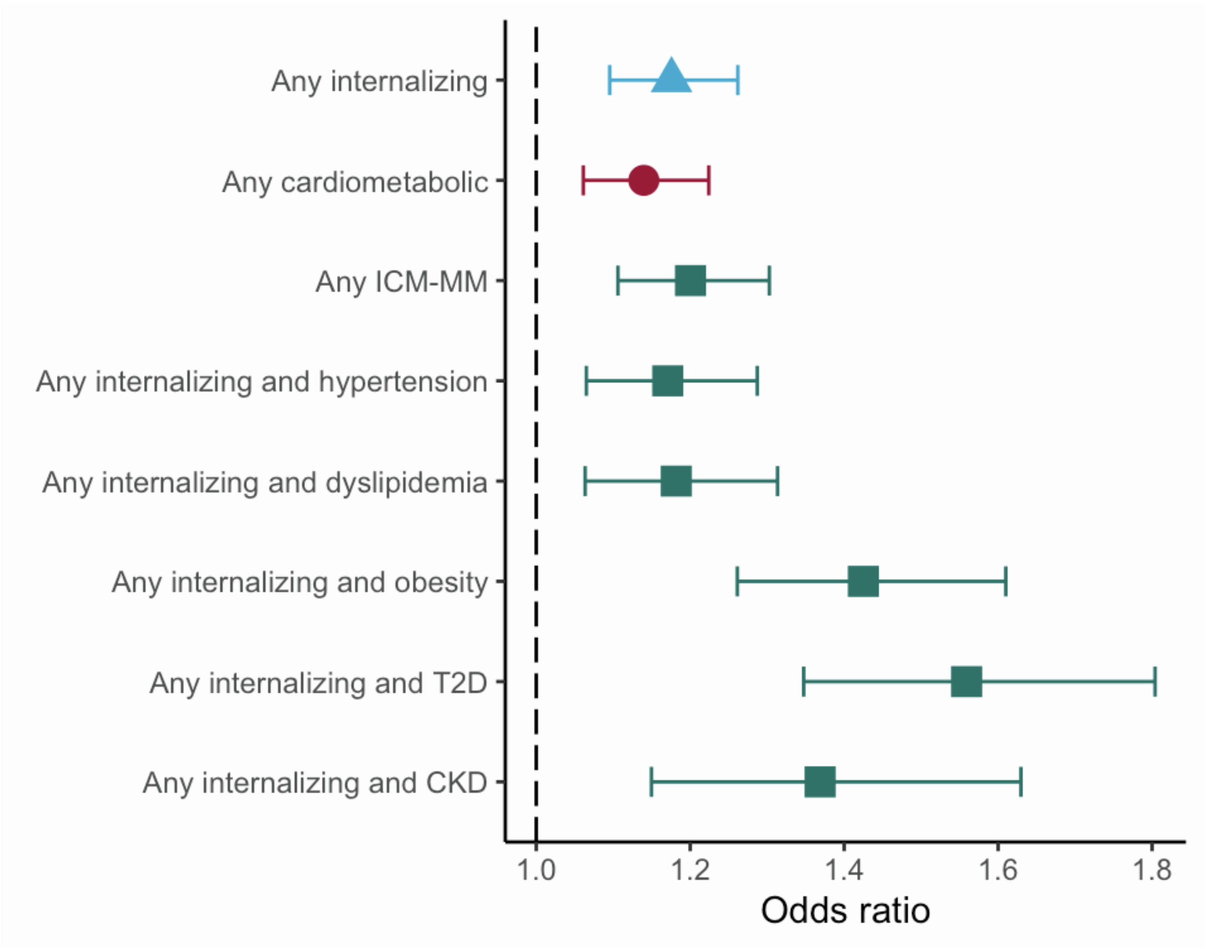

Figure S2. Association of ND-CNVs with multimorbidity excluding individuals with 16p11.2 proximal and distal deletions (N= 185).

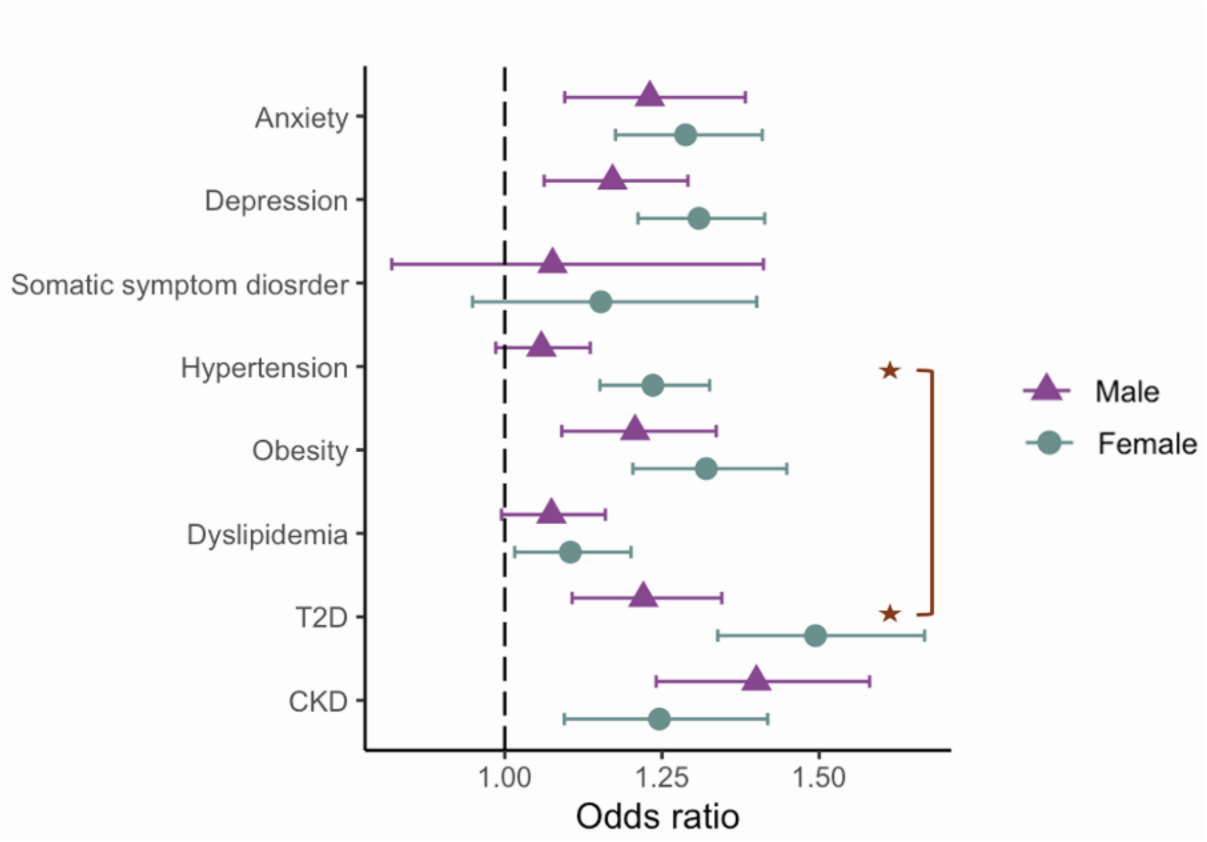

Figure S3. Association of ND-CNVs with internalizing and cardiometabolic conditions for male and female sex. Stars indicate a significant interaction of presence of ND-CNV and sex.

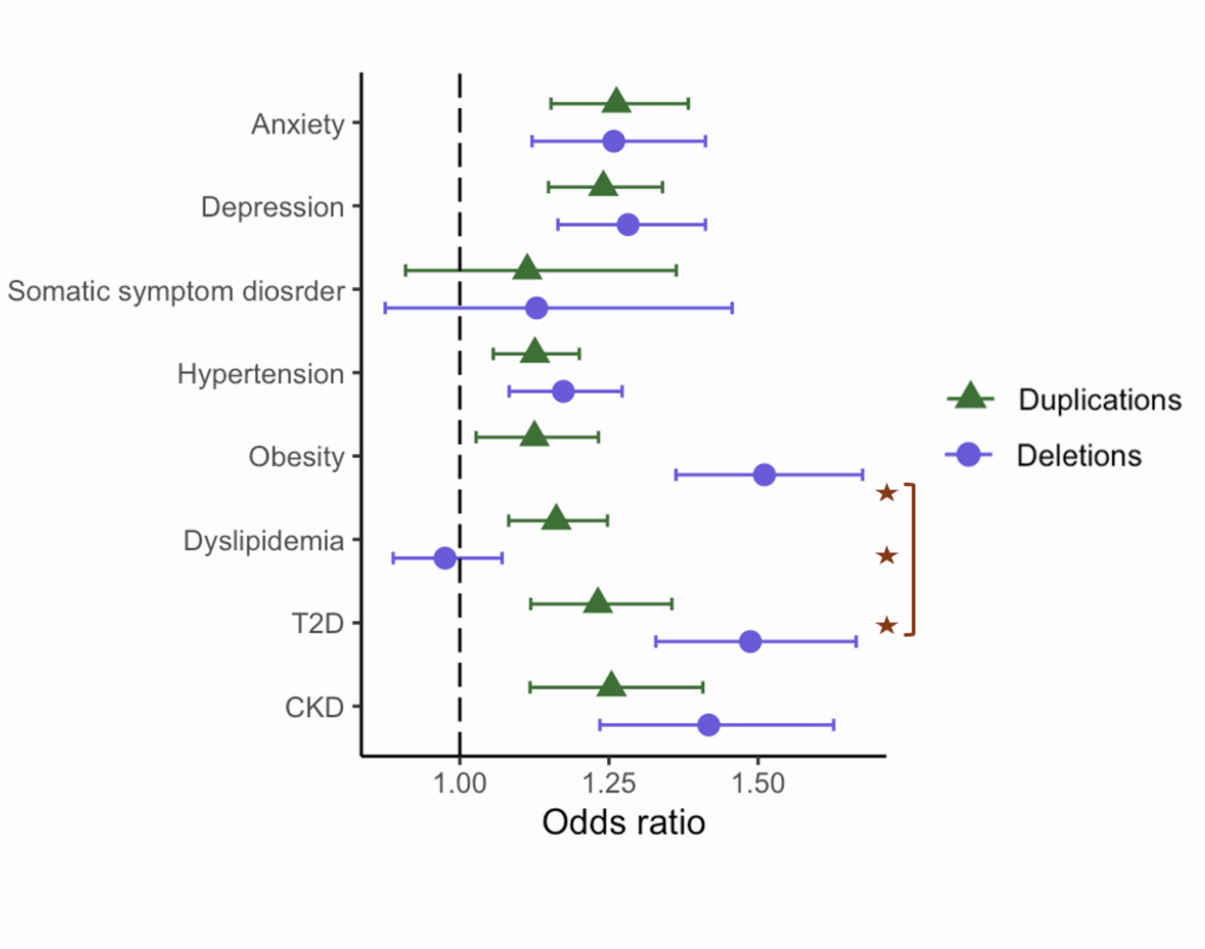

Figure S4. Association of duplications and deletions with internalizing and cardiometabolic conditions. Stars indicate a suggested difference between deletions and duplications (p-value < 0.05).

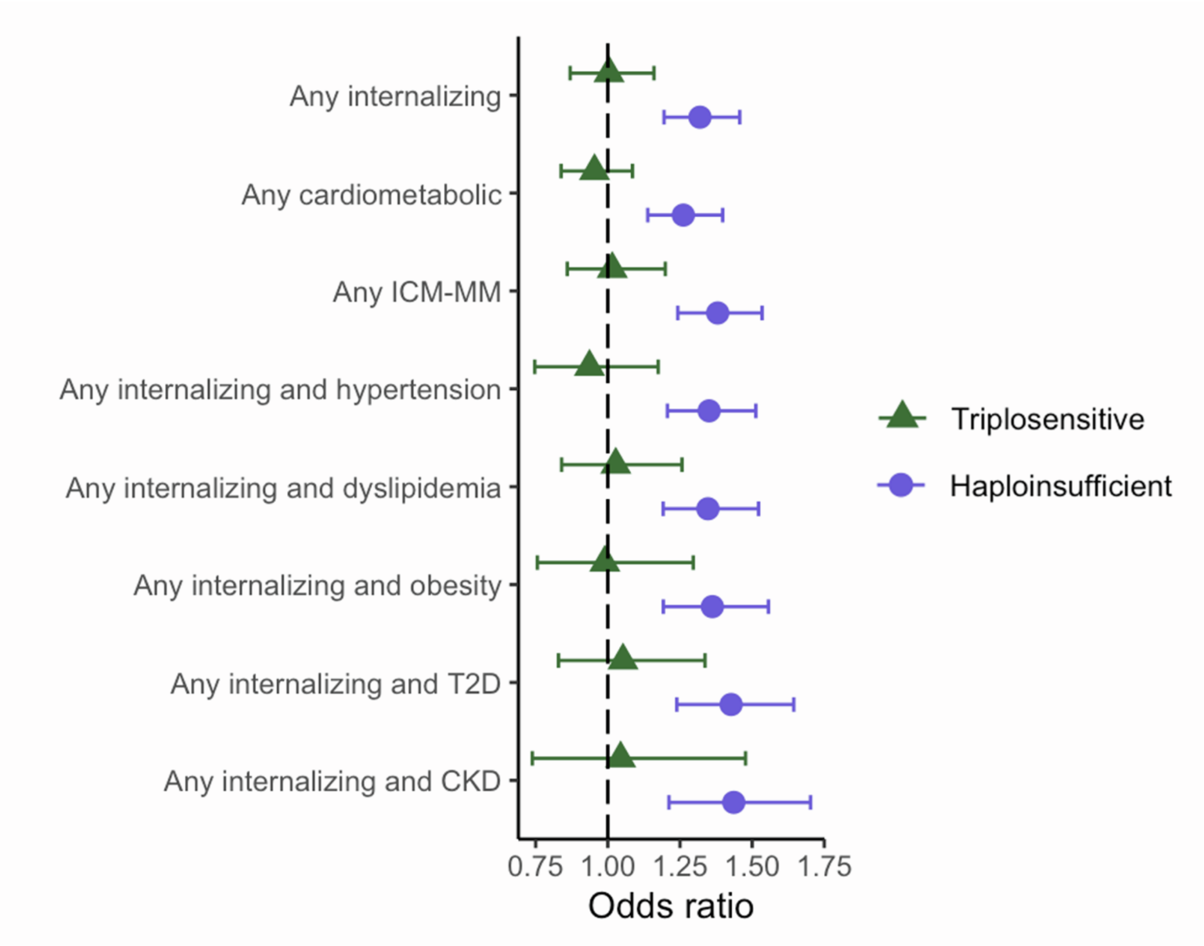

Figure S5. Association of triplosensitive genes in duplications and haploinsufficient genes in deletions with ICM-MM. ORs represent an increase in risk by an increase of one gene.

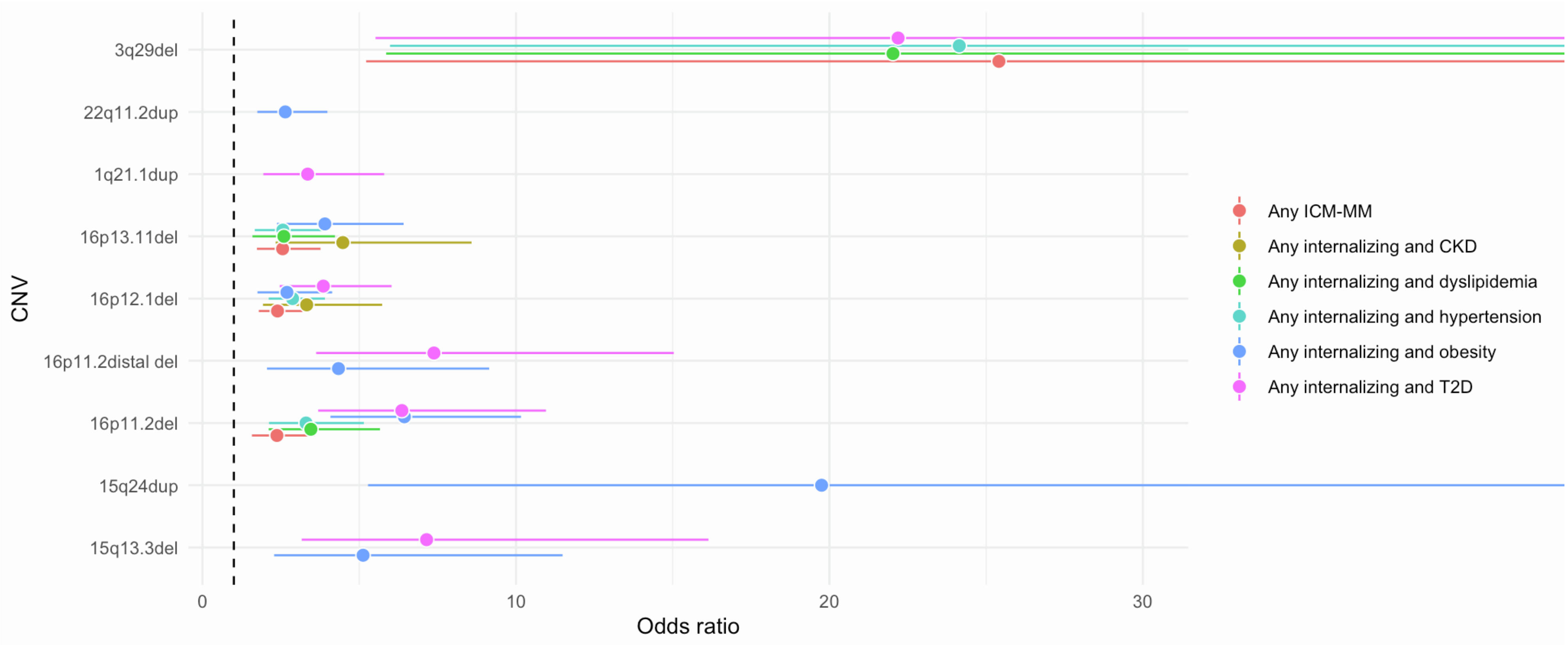

Figure S6. Association of individual ND-CNVs and multimorbidity. Only significant associations after Bonferroni correction for multiple testing are included in the figure.

SUPPLEMENTAL TABLES

Trait	GWAS
MDD	Wray *et al.*, 2018 (UKBB removed) (3)
Anxiety	Meier *et al.*, 2019 (4)
LDL	Willer *et al.*, 2013 (5)
SBP	Keaton *et al.*, 2024 (6) (only ICBP)
BMI	Locke *et al*. 2015 (7)
T2D	Mahajan *et al.*, 2018 (UKBB removed) (8)
CKD	Pattaro *et al.*, 2016 (9)

Table S1. Genome-wide association studies used for polygenic risk score generation.

CNV	Count
Any	7,546
1p36 del	1
TAR del	80
TAR dup	463
1q21.1 del	119
1q21.1 dup	193
NRXN1 del	176
2q11.2 del	34
2q13 del	56
2q13 dup	73
2q37 del	1
3q29 del	9
WH dup	3
WBS del	1
WBS dup	16
8p23.1 del	4
8p23.1 dup	8
EMHT1 dup	1
10q23 del	3
15q11.2 del	1,748
15q11.2 dup	2,284

PWS del	1
PWS dup	19
15q13.3 del	47
15q24 del	1
15q24 dup	9
15q25 del	1
16p13.11 del	140
16p13.11 dup	888
16p12.1 del	260
16p11.2distal del	62
16p11.2distal dup	142
16p11.2 del	123
16p11.2 dup	142
17p13.3 YWHAE del	27
17p13.3 YWHAE dup	8
17p13.3 PAFAH1B1 del	1
17p13.3 PAFAH1B1 dup	3
SMS	2
Potocki Lupski	6
17q11.2 delNF1	10
17q11.2 dup NF1	3
17q12 del	9
17q12 dup	104
22q11.2 del	10
22q11.2 dup	294
22q11.2 distal del	5
22q11.2 distal dup	14

Table S2. Number of individuals with each ND-CNV. ND-CNVs that appeared less than five times are in italics.

	ND-CNV	No ND-CNV
Age at recruitment (Mean (min, max))	56.07 (40,70)	56.55 (37,73)
Townsend deprivation index (Mean (min, max))	-0.86 (-6.26, 10.56)	-1.32 (-6.26, 11.00)
Female (N (%))	3,934 (52.14)	245,589 (54.32)

Table S3. Demographic characteristics of individuals with and without a ND-CNV. NV. Townsend deprivation index is measure of socioeconomic deprivation, with higher value indicating a more deprived environment.

	ND-CNV	(N=7,546)	No ND-CNV	(N=451,937)
	Cases	Frequency (%)	Cases	Frequency (%)
Anxiety	878	11.6	42,365	9.4
Depression	1,335	17.7	65,040	14.4
Somatic symptom disorder	161	2.1	8,513	1.9
Any internalizing disorder	1,685	22.3	84,755	18.8
Hypertension	2,765	36.6	153,829	34.0
Obesity	975	12.9	46,176	10.2
Chronic kidney disease	570	7.6	26,924	6.0
Type II diabetes	870	11.5	39,236	8.7
Dyslipidemia	1,709	22.6	95,960	21.2
Any CM	3,705	49.1	206,240	45.7

Table S4. Counts and frequency for each of the conditions of interest in individuals with and without a ND-CNV.

	OR (CI)	p-value
Anxiety	1.25 (1.16-1.34)	9.16×10^{-10}
Depression	1.24 (1.16-1.31)	5.11×10^{-12}
Somatic symptom disorder	1.13 (0.96-1.32)	0.144
Any internalizing condition	1.22 (1.15 -1.28)	4.50×10^{-12}
Hypertension	1.16 (1.10-1.22)	1.20×10^{-8}
Obesity	1.27 (1.18-1.36)	1.04×10^{-11}
Dyslipidaemia	1.10 (1.22-1.46)	1.69×10^{-3}
Type II diabetes	1.37 (1.27-1.48)	2.43×10^{-17}
Chronic kidney disease	1.33 (1.03-1.16)	1.45×10^{-10}
Any cardiometabolic condition	1.19 (1.13-1.24)	5.37×10^{-12}
Any internalizing and hypertension	1.21 (1.13-1.31)	5.55×10^{-7}
Any internalizing and dyslipidemia	1.27 (1.16-1.38)	1.34×10^{-7}
Any internalizing and obesity	1.48 (1.33-.164)	1.51×10^{-13}
Any internalizing T2D	1.57 (1.38-1.77)	1.66×10^{-6}
Any internalizing and CKD	1.44 (1.24-1.66)	1.09×10^{-6}
Any ICM-MM	1.25 (1.17-1.34)	3.14×10^{-11}

Table S5. Association of individual conditions and ICM-MM phenotypes with the presence of a ND-CNV.

	OR (CI)	p-value
Any ICM-MM	1.18 (1.10-1.26)	1.32×10^{-6}
Any internalizing and hypertension	1.14 (1.05-1.23)	9.56×10^{-4}
Any internalizing and dyslipidemia	1.20 (1.10-1.32)	3.89×10^{-5}
Any internalizing T2D	1.41 (1.24-1.60)	7.11×10^{-8}
Any internalizing and CKD	1.35 (1.17-1.56)	6.53×10^{-5}

Table S6. Association of ICM-MM phenotypes with the presence of a ND-CNV adjusting for BMI. Obesity was not included as an outcome in this analysis.

	OR (CI)	p-value
Any ICM-MM	1.20 (1.11-1.30)	$1.21x10^{-5}$
Any internalizing and hypertension	1.17 (1.07-1.29)	$1.09x10^{-3}$
Any internalizing and dyslipidemia	1.18 (1.06-1.31)	$1.92x10^{-3}$
Any internalizing and obesity	1.48 (1.26-1.61)	$1.30x10^{-8}$
Any internalizing T2D	1.56 (1.35-1.80)	$2.42x10^{-9}$
Any internalizing and CKD	1.37 (1.15-1.63)	$4.20x10^{-4}$

Table S7. Association of ICM-MM phenotypes with the presence of a ND-CNV for individuals with both primary care and HES data (N= 229,951).

	OR (CI)	p-value
Any ICM-MM	1.20 (1.11-1.30)	$2.08x10^{-9}$
Any internalizing and hypertension	1.17(1.07-1.29	$7.64x10^{-6}$
Any internalizing and dyslipidemia	1.18 (1.06-1.31)	$3.84x10^{-6}$
Any internalizing and obesity	1.42 (1.26-1.61)	$8.95x10^{-10}$
Any internalizing T2D	1.56 (1.35-1.80)	$7.06x10^{-10}$
Any internalizing and CKD	1.37 (1.15-1.63)	$4.15x10^{-6}$

Table S8. Association of ICM-MM phenotypes with the presence of a ND-CNV excluding individuals with 16p11.2 proximal and distal deletions (N= 185).

	White		Black		Asian		Mixed		Other	
	N = 432,604		N = 7,170		N = 10,194		N = 2,626		N = 4,095	
	OR (CI)	p	OR (CI)	p	OR (CI)	p	OR (CI)	p	OR (CI)	p
Any ICM-MM	1.25 (1.17-1.34)	4.20×10^{-11}	1.64 (0.80-3.37)	0.171	1.37 (0.65-4.98)	0.191	0.86 (0.26-2.90)	0.815	1.70 (0.17-1.83)	0.330
Any internalizing and hypertension	1.21 (1.12-1.31)	1.42×10^{-6}	1.67 (0.75-3.71)	0.206	1.47 (0.87-2.49)	0.142	0.87 (0.202-3.71)	0.846	0.76 (0.23-2.56)	0.678
Any internalizing and dyslipidemia	1.28 (1.18-1.40)	3.59×10^{-8}	3.99 (0.05-2.91)	0.364	1.03 (0.55-1.94)	0.916	1.31 (0.303-5.71)	0.713	0.30 (0.04-2.19)	0.235
Any internalizing and obesity	1.48 (1.33-1.64)	4.85×10^{-13}	1.78 (0.64-5.01)	0.271	1.14 (0.46-2.84)	0.777	2.47 (0.72-8.48)	0.150	0.02 (0-inf)	0.970
Any internalizing and T2D	1.57 (1.39-1.78)	1.04×10^{-12}	1.42 (0.43-4.60)	0.557	1.82 (1.01-3.29)	0.045	8.71 (0-inf)	0.983	1.05 (0.25-4.29)	0.940
Any internalizing and CKD	1.46 (1.26-1.69)	5.29×10^{-7}	1.61 (0-inf)	0.977	1.80 (0.64-4.98)	0.260	1.75 (0-inf)	0.983	0.01 (0-inf)	0.981

Table S9. Association of ICM-MM phenotypes with the presence of a ND-CNV stratified by ethnicity.

	Male sex	Female sex	Difference
	**OR (CI)**	**OR (CI)**	**p-value**
Any internalizing	1.20 (1.10-1.31)	1.25 (1.16-1.34)	0.512
Any cardiometabolic	1.09 (1.02-1.17)	1.25 (1.17-1.34)	0.004
Any ICM-MM	1.19 (1.08-1.32)	1.31 (1.20-1.43)	0.211
Any internalizing and hypertension	1.12 (0.99-1.26)	1.31 (1.18-1.45)	0.063
Any internalizing and dyslipidemia	1.19 (1.05-1.36)	1.34 (1.19-1.51)	0.214
Any internalizing and obesity	1.39 (1.17-1.66)	1.55 (1.36-1.76)	0.328
Any internalizing and T2D	1.35 (13.1-1.61)	1.78 (1.51-2.10)	0.026
Any internalizing and CKD	1.61 (1.30-1.99)	1.32 (1.08-1.62)	0.181

Table S10. Association of ICM-MM phenotypes with ND-CNV for male and female sex.

	Duplications	Deletions	Difference
	**OR (CI)**	**OR (CI)**	**p-value**
Any internalizing	1.21 (1.13-1.29)	1.26 (1.16-1.38)	0.834
Any cardiometabolic	1.17 (1.10-1.24)	1.17 (1.08-1.26)	0.980
Any ICM-MM	1.21 (1.11-1.32)	1.32 (1.19-1.47)	0.430
Any internalizing and hypertension	1.16 (1.05-1.28)	1.31 (1.17-1.48)	0.281
Any internalizing and dyslipidemia	1.31 (1.18-1.47)	1.20 (1.04-1.38)	0.596
Any internalizing and obesity	1.27 (1.10-1.46)	1.85 (1.59-2.15)	0.002
Any internalizing and T2D	1.45 (1.24-1.70)	1.72 (1.44-2.07)	0.267
Any internalizing and CKD	1.34 (1.11-1.62)	1.61 (1.29-2.02)	0.439

Table S11. Association of ICM-MM phenotypes with duplications and deletions.

	Deletions		Duplications	
	**Haploinsufficient genes**	**Total genes**	**Triplosensitive genes**	**Total genes**
	**OR (CI)**	**OR (CI)**	**OR (CI)**	**OR (CI)**
Any internalizing	1.31 (1.19-1.46) *	1.01 (1.00-1.01) *	1.01 (0.86-1.15)	1.01 (1.00-1.01) *
Any cardiometabolic	1.26 (1.14-1.40) *	1.01 (1.00-1.01) *	0.95 (0.84-1.08)	1.01 (1.00-1.01) *
Any ICM-MM	1.38 (1.24-1.53) *	1.01 (1.00-1.01) *	1.01 (0.86 -1.20)	1.01 (1.00-1.01) *
Any internalizing and hypertension	1.35 (1.21-1.51) *	1 (1.00-1.01)	0.94 (0.75-1.17)	1. 01 (1.00-1.01)
Any internalizing and dyslipidemia	1.35 (1.19-1.52) *	1.01 (1.00-1.01) *	1.03 (0.84-1.25)	1.01 (1.00-1.01) *
Any internalizing and obesity	1.36 (1.10-1.55) *	1.01 (1.01-1.03) *	0.99 (0.76-1.30)	1.01 (1.00-1.01) *

Any internalizing and T2D	1.43 (1.24.1.64) *	1.01 (1.01-1.02) *	1.05 (0.83-1.34)	1.01 (1.00-1.01) *
Any internalizing and CKD	1.44 (1.21-1.70) *	1.01 (1.00-1.01)	1.04 (0.74-1.48)	1.01 (1.00-1.01)

Table S12. Association of haploinsufficient and total genes in deletions and triplosensitive and total genes in duplications with ICM-MM. ORs represent odds by an increase of one gene. Asterisks indicate statistical significance after Bonferroni correction for multiple testing.

	ND-CNV*PRS													
	MDD		Anxiety		T2D		BMI		SBP		LDL		CKD	
**Outcome**	**OR (CI)**	**p-value**	**OR (CI)**	**p-value**	**OR (CI)**	**p-value**	**OR (CI)**	**p-value**	**OR (CI)**	**p-value**	**OR (CI)**	**p-value**	**OR (CI)**	**p-value**
**Any ICM-MM**	1.01 (0.94, 1.08)	0.857	1.03 (0.96,1.10)	0.438	1.00 (0.93,1.08)	0.983	0.98 (0.91,1.06)	0.668	0.96 (0.89,1.03)	0.269	1.03 (0.96,1.11)	0.351	1.02 (0.95,1.09)	0.606
**Any internalizing and hypertension**	0.96 (0.8, 81.04)	0.323	1.01 (0.93,1.09)	0.842	1.02 (0.94,1.11)	0.616	1.01 (0.92,1.10)	0.902	0.98 (0.90,1.07)	0.678	1.06 (0.97,1.14)	0.183	1.01 (0.93,1.10)	0.775
**Any internalizing and dyslipidemia**	1.04 (0.94, 1.14)	0.468	1.07 (0.97,1.17)	0.176	1.01 (0.92,1.12)	0.795	0.97 (0.87,1.07)	0.500	1.02 (0.93,1.12)	0.651	1.05 (0.95,1.15)	0.332	1.03 (0.94,1.13)	0.535
**Any internalizing and obesity**	1.10 (0.98, 1.23)	0.094	1.15 (1.04,1.28)	0.010	0.97 (0.87,1.09)	0.642	0.98 (0.87,1.10)	0.738	1.02 (0.92,1.15)	0.667	1.02 (0.91,1.13)	0.748	1.07 (0.96,1.19)	0.237
**Any internalizing and T2D**	0.93 (0.82, 1.07)	0.317	1.01 (0.89,1.14)	0.884	1.01 (0.88,1.16)	0.907	0.98 (0.85,1.13)	0.775	1.09 (0.96,1.25)	0.177	1.05 (0.93,1.20)	0.428	0.95 (0.84,1.08)	0.413
**Any internalizing and CKD**	1.06 (0.90, 1.24)	0.486	1.18 (1.01,1.37)	0.037	0.88 (0.75,1.04)	0.143	0.94 (0.79,1.11)	0.470	1.05 (0.90,1.23)	0.549	1.18 (1.01,1.37)	0.039	1.01 (0.87,1.18)	0.893

Table S13. Interaction between presence of ND-CNVs and PRSs on outcome risk. To assess whether the association of common genetic variation and the risk of multimorbidity differs in individuals with and without a ND-CNV, logistic regression analyses were performed for each of the outcomes, including the main effects of each ancestry-adjusted PRS, ND-CNV and the interaction term PRS*ND-CNV. The odds ratios and p-values correspond to the ND-CNV*PRS interaction term for each of the seven PRSs examined.

PRS	OR (CI)	p-value
MDD	1.05 (1.02-1.08)	1.21×10^{-4}
Anxiety	1.06 (1.03-1.08)	2.21×10^{-6}
T2D	1.01 (0.99-1.04)	0.296
BMI	1.02 (1.00-1.05)	0.087
SBP	1.01 (0.99-1.04)	0.344
LDL	1.00 (0.97-1.02)	0.980
CKD	1.04 (1.01-1.06)	2.52×10^{-3}

Table S14. Association of ND-CNV with each of the PRSs.

SUPPLEMENTAL REFERENCES

1. Eto F, Samuel M, Finer S. MULTIPLY-Initiative: Version 1.1 [Internet]. Zenodo; 2023 [cited 2023 Aug 31]. Available from: https://zenodo.org/record/7643566
2. Baksh RA, Pape SE, Chan LF, Aslam AA, Gulliford MC, Strydom A. Multiple morbidity across the lifespan in people with Down syndrome or intellectual disabilities: a population-based cohort study using electronic health records. Lancet Public Health. 2023 Jun 1;8(6):e453–62.
3. Wray NR, Ripke S, Mattheisen M, Trzaskowski M, Byrne EM, Abdellaoui A, et al. Genome-wide association analyses identify 44 risk variants and refine the genetic architecture of major depression. Nat Genet. 2018 May;50(5):668–81.
4. Meier SM, Trontti K, Purves KL, Als TD, Grove J, Laine M, et al. Genetic Variants Associated With Anxiety and Stress-Related Disorders: A Genome-Wide Association Study and Mouse-Model Study. JAMA Psychiatry. 2019 Sep 1;76(9):924–32.
5. Willer CJ, Schmidt EM, Sengupta S, Peloso GM, Gustafsson S, Kanoni S, et al. Discovery and Refinement of Loci Associated with Lipid Levels. Nat Genet. 2013 Nov;45(11):1274.
6. Keaton JM, Kamali Z, Xie T, Vaez A, Williams A, Goleva SB, et al. Genome-wide analysis in over 1 million individuals of European ancestry yields improved polygenic risk scores for blood pressure traits. Nat Genet. 2024 May;56(5):778–91.
7. Locke AE, Kahali B, Berndt SI, Justice AE, Pers TH, Day FR, et al. Genetic studies of body mass index yield new insights for obesity biology. Nature. 2015 Feb;518(7538):197–206.
9. Mahajan A, Taliun D, Thurner M, et al. Fine-mapping type 2 diabetes loci to single-variant resolution using high-density imputation and islet-specific epigenome maps. Nat Genet. 2018;50(11):1505-1513. doi:10.1038/s41588-018-0241-6
10. Pattaro, C., Teumer, A., Gorski, M. et al. Genetic associations at 53 loci highlight cell types and biological pathways relevant for kidney function. Nat Commun 7, 10023 (2016). https://doi.org/10.1038/ncomms10023
