# Supplementary material for: Neurodevelopmental copy-number variants increase risk of internalizing and cardiometabolic multimorbidity: Findings from the UK Biobank
Source: Am J Hum Genet. 2026 Apr 6;113(6):1319–29. doi: 10.1016/j.ajhg.2026.02.021 (PMC13277688; doi:10.1016/j.ajhg.2026.02.021)
Supplement: Document S2. Article plus supplemental information [file mmc3.pdf]

Neurodevelopmental copy-number variants increase risk of internalizing and cardiometabolic multimorbidity: Findings from the UK Biobank

Authors

Ioanna K. Katzourou, LINC consortium,
Inês Barroso, ..., Michael J. Owen,
Peter Holmans, Marianne B.M. van den Bree

Correspondence

vandenbreemb@cardiff.ac.uk

We show that neurodevelopmental copy-number variants increase the risk of internalizing-cardiometabolic multimorbidity in an older-age population-based cohort. This highlights the clinical relevance of rare genomic variation for multimorbidity risk. Effects are stronger in females and for deletions compared to duplications and appear to be independent of polygenic risk.

Katzourou et al., 2026, The American Journal of Human Genetics *113*, 1319–1329

June 4, 2026 © 2026 The Author(s). Published by Elsevier Inc. on behalf of American Society of Human Genetics.

https://doi.org/10.1016/j.ajhg.2026.02.021

Neurodevelopmental copy-number variants increase risk of internalizing and cardiometabolic multimorbidity: Findings from the UK Biobank

Ioanna K. Katzourou,[1] LINC consortium, Inês Barroso,[2] Julie Clayton,[3] Golam Khandaker,[4] Daniel Stow,[5] Nicolas Timpson,[4] Ruby Tsang,[4] Jack Underwood,[1] Megan Wood,[6] George Kirov,[1] James Walters,[1] Michael J. Owen,[1,7] Peter Holmans,[1] and Marianne B.M. van den Bree[1,7,*]

Summary

Internalizing and cardiometabolic multimorbidity (ICM-MM) represents a major clinical challenge, negatively impacting life expectancy and quality of life and resulting in considerable healthcare costs. Individuals with a copy-number variant associated with increased risk of neurodevelopmental conditions (ND-CNV) are more likely to develop mental or physical ill health; however, the effects on ICM-MM remain poorly understood. We used data from the UK Biobank (ND-CNV $N = 7{,}549$, 1.62%) to examine the effect of ND-CNVs on ICM-MM. ICM-MM was defined as a combination of any internalizing condition (depression, anxiety, or somatic symptom disorder) with each of five cardiometabolic conditions (hypertension, dyslipidemia, obesity, type 2 diabetes (T2D), and chronic kidney disease). We also studied whether ICM-MM risk in those with ND-CNVs differed by sex or presence of a deletion versus a duplication. We established associations between dosage-sensitive genes within ND-CNVs and ICM-MM and explored the interaction between the presence of ND-CNVs and polygenic risk scores (PRSs) of internalizing and cardiometabolic traits on ICM-MM risk. The presence of ND-CNVs was associated with ICM-MM (odds ratio [OR] range: 1.21–1.57). Female participants with ND-CNVs were more likely to have any internalizing condition and T2D, and those with a deletion were more likely to have any internalizing condition and obesity. The number of deleted haploinsufficient genes, but not duplicated triplosensitive genes, was associated with ICM-MM. No interactions between ND-CNVs and PRSs were found. We find that ND-CNVs increase the likelihood of ICM-MM, with evidence of sex differences and stronger effects for deletions. Increased clinical awareness can help ameliorate this risk.

Introduction

Multimorbidity, also referred to as multiple long-term conditions (MLTCs), indicates the presence of two or more chronic health conditions in the same individual. Multimorbidity represents a major public health concern, with at least 50 million people affected in the European Union alone.[1] An estimated 25% of the population of high-income countries is living with two or more conditions, and rates are rapidly increasing in low- and middle-income countries.[1,2] Multimorbidity is associated with high personal and societal healthcare costs.[2] Moreover, multimorbidity is difficult to manage, resulting in a high risk of failure of care and putting considerable strain on healthcare systems.[1–3]

Cardiometabolic conditions, such as hypertension, obesity, and type 2 diabetes (T2D), and internalizing conditions, such as depression and anxiety, are highly prevalent, affecting millions of people worldwide and resulting in increased disability and functional decline, poor quality of life, and premature mortality.[4,5] The co-occurrence of internalizing and cardiometabolic conditions represents the most common type of physical and mental health multimorbidity in older adults.[3] While both cardiometabolic and internalizing conditions pose considerable public health challenges, their presence in combination is particularly burdensome for the individuals affected.[4,5] Internalizing disorders are associated with an increased risk of subsequent cardiometabolic disorders[6–8] as well as cardiovascular morbidity and premature mortality.[9,10] The reverse is also the case, with those with cardiometabolic conditions experiencing more depression and anxiety.[7,10–12] Importantly, the lower life expectancy associated with depression[13] is partially attributable to comorbid cardiovascular disease.[14] These findings highlight the importance of uncovering the risk factors contributing to internalizing and cardiometabolic multimorbidity (ICM-MM).

While the exact mechanisms leading to the development of ICM-MM are unknown, shared genetic risk factors may play a considerable role.[15,16] Polygenic risk scores (PRSs) of cardiometabolic disorders have been found to be associated with depression,[17,18] while high genetic overlap has also been found between depression and T2D.[19] Most published studies focus on common genetic variation, while the effect of rare genetic variants on

[1]Centre for Neuropsychiatric Genetics and Genomics, Cardiff University, Cardiff, UK; [2]Medical School, University of Exeter, Exeter, UK; [3]Centre for Academic Primary Care, University of Bristol, Bristol, UK; [4]Bristol Medical School, University of Bristol, Bristol, UK; [5]Wolfson Institute of Population Health, Queen Mary University of London, London, UK; [6]School of Psychology, University of Leeds, Leeds, UK; [7]Neuroscience and Mental Health Innovation Institute, Cardiff University, Cardiff, UK
*Correspondence: vandenbreemb@cardiff.ac.uk
https://doi.org/10.1016/j.ajhg.2026.02.021.
© 2026 The Author(s). Published by Elsevier Inc. on behalf of American Society of Human Genetics.
This is an open access article under the CC BY license (http://creativecommons.org/licenses/by/4.0/).

multimorbidity remains mostly unexplored. Copy-number variants (CNVs) are structural alterations in chromosomes involving the deletion or duplication of a section of varying length.[20] A range of CNVs increases the risk of neurodevelopmental conditions (NDCs), such as intellectual disability, autism spectrum disorder, attention-deficit hyperactivity disorder, and schizophrenia.[21–24] These are referred to as CNVs associated with increased risk of neurodevelopmental conditions (ND-CNVs).[25] In addition to NDCs, these CNVs can also increase the risk of internalizing conditions[26] as well as a range of physical health conditions and traits,[27] including hypertension, diabetes, and body mass index (BMI). There is increasing evidence of increased risk of cardiovascular morbidity in individuals with some of these variants.[28–30] As ND-CNVs are often diagnosed in childhood, increased understanding of the development of multimorbidity in individuals with ND-CNVs presents opportunities for a better understanding of the development of disease development and potentially also provides important insights into the biological mechanisms of multimorbidity.

In this study, we explored the association between ICM-MM and ND-CNVs[21] in the UK Biobank (UKBB)[31] (www.ukbiobank.ac.uk).

The objectives of this study were to

(1) Determine the association of ND-CNVs, loss (deletion) and gain (duplication) of chromosomal material, and dosage-sensitive genes in ND-CNVs with ICM-MM;
(2) Investigate sex differences in the effect of ND-CNVs on ICM-MM; and
(3) Establish if the effect of common variation (PRSs of internalizing and cardiometabolic traits) on ICM-MM differs in individuals with versus without an ND-CNV.

Subjects and methods

Data source

The UKBB is a prospective cohort of over 500,000 individuals living in the United Kingdom.[31] The UKBB received ethical approval from the North West-Haydock Research Ethics Committee (ref. 16/NW/0274). Participants provided electronically signed consent at recruitment. This study was conducted under application number 79704.

Participants

Participants aged between 40 and 69 years old were recruited into the UKBB between 2006 and 2010.[31] Sociodemographic, lifestyle, and medical history information were collected using touchscreen questionnaires. Physical and functional measurements, biochemical assays, and genome-wide genotyping were collected at a baseline assessment. Linkage to the National Health Service provided data on deaths, cancer diagnoses, hospital inpatient/outpatient episodes, and primary care records. Details of the UKBB study design are provided elsewhere.[31] Out of the total number of UKBB participants (502,411), 459,483 (91.47%) had available CNV call data and were included in this study.

Phenotyping

This study took place under the Lifespan Multimorbidity Research Collaborative (LINC) (https://www.cardiff.ac.uk/lifespan-multimorbidity-research-collaborative). LINC seeks to understand the development of ICM-MM over the life course. The conditions included in LINC's definition of ICM-MM were selected following discussions with primary and secondary care doctors, the LINC patient and public involvement team, and the LINC team of researchers and clinicians.

Three internalizing conditions were included: depression, anxiety, and somatic symptom disorder. Five cardiometabolic conditions were included: hypertension, dyslipidemia, obesity, T2D, and chronic kidney disease (CKD). The cardiometabolic conditions included in the definition are restricted to those that generally occur earlier in life than established cardiovascular disease, allowing the opportunity to study the increase in risk over an extended period.

Individuals with each of the conditions of interest were identified through linked electronic healthcare records (EHRs; primary care records and hospital episode statistics [HESs]) using established clinical codelists (see the supplemental information). We examined the pairwise combinations of aggregated internalizing conditions, defined as the presence of one or more of the three internalizing conditions listed above (referred to as "any internalizing condition" from here onwards), and each of the five cardiometabolic conditions. We also constructed an outcome variable—any ICM-MM—defined as the presence of one or more of the three internalizing conditions and one or more of the five cardiometabolic conditions.

Defining ND-CNVs

The calling of ND-CNVs in the UKBB had previously been performed and is described in detail elsewhere.[32] Briefly, calling was performed using PennCNV-Affy 1.0.3 protocols[33] on the UKBB genotype array data. Affymetrix Power Tools software (www.affymetrix.com/estore/partners_programs/programs/developer/tools/powertools.affx) was used to generate signal intensity data, genotype calls, and confidences, which were then processed with the PennCNV-Affy software. Predefined batches were processed separately to reduce potential batch effects. Adjacent CNVs were joined if separated by less than 25% of their total combined length. Samples were excluded if they carried 30 or more CNVs or had a waviness factor greater than 0.03 or less than −0.03, a single-nucleotide polymorphism call rate lower than 96%, or a log R ratio SD higher than 0.35. CNVs were excluded if they were covered by fewer than 20 probes or had a density coverage of less than 1 probe per 20,000 base pairs or a confidence score lower than 10. This resulted in 459,483 individuals with available CNV call data. The breakpoints of a list of 93 CNVs proposed to be pathogenic (which include the ND-CNVs analyzed in this study) were visually inspected to validate that they met the calling criteria: we required the CNV to cover more than half the critical interval, including known key genes in the region, or, in the case of single-gene CNVs, to intersect at least one exon (for deletions) or to cover the whole gene (for duplications).

Statistical analyses

All statistical analyses were performed in R.[34]

Aggregated CNVs

We focused on a set of 54 CNVs that show strong evidence of increasing the risk of developing a neurodevelopmental disorder

(ND-CNVs).[21] We assessed the association of the presence of any ND-CNV (aggregated ND-CNV; binary variable) with each of the conditions of interest individually, as well as pairwise combinations of any internalizing conditions and each cardiometabolic condition and any ICM-MM (binary variables). We used logistic regression of each of these phenotypes on aggregated ND-CNVs, adjusting for age at baseline, sex, and Townsend deprivation index (as a measure of socioeconomic status), as well as the first five genetic principal components to account for population stratification. To avoid overestimating associations due to the low frequency of ND-CNVs, Firth's bias correction method[35] was performed for the regression models. This method always leads to finite parameter estimates, unlike the maximum likelihood method normally used in regression models, and can therefore avoid separation even with small samples. The logistf() R package was used for the correction (https://github.com/georgheinze/logistf).

Sensitivity analyses

While HES data are available for the whole UKBB cohort ($N = 502{,}390$), primary care records are available for ~40% of the participants ($N = 229{,}951$). A sensitivity analysis was performed, including only participants for whom both HES and primary care records were available ($N = 229{,}951$).

Deletions in the 16p11.2 region have been previously associated with class III obesity (formerly known as morbid obesity).[36–38] Obesity is one of the five cardiometabolic conditions we examined and is a known risk factor for the remaining four. In order to determine if the overall effect of ND-CNVs on ICM-MM was driven by these specific ND-CNVs, we repeated the regression analysis after removing individuals with a proximal or distal deletion of 16p11.2 ($N = 185$). We also repeated the regressions, adjusting for the BMI at baseline (excluding obesity as an outcome) to account for possible effects of the ND-CNVs on body mass.

In order to assess if the results differed by ethnicity, we repeated the regression analyses while stratifying participants by ethnicity, using the self-reported groups provided by the UKBB (White, Black or Black British, Asian or Asian British, mixed, Chinese, and other; field 21000). Due to the low number of participants ($N = 1{,}573$) who self-reported as Chinese, this group was included in the Asian ethnicity.

Post hoc analyses

To assess if there are any sex differences in the associations described above, we also conducted the above regression including an interaction term between ND-CNV and sex. Moreover, we also assessed the association of the phenotypes with the type of NDD-CNV, e.g., loss (deletion) or gain (duplication). For this analysis, genotype was coded as a three-level variable (no ND-CNV, deletion, or duplication). Logistic regressions of each of the phenotypes on genotype were performed, adjusting for age, sex, Townsend deprivation index, and the first five genetic principal components. Individuals with both a deletion and a duplication were excluded from this analysis ($N = 12$).

We next examined whether dosage-sensitive genes within ND-CNVs are associated with ICM-MM. The coordinates for each ND-CNV were used to map which genes were affected in each individual. Out of the genes affected by an ND-CNV in each individual, we identified those that are haploinsufficient (deletion intolerant) or triplosensitive (duplication intolerant), based on a dosage sensitivity analysis conducted by Collins et al.,[39] and calculated the number of dosage-sensitive genes affected in each individual. For this analysis, deletions and duplications were analyzed separately. Thus, we regressed the phenotypes of interest (e.g., pairwise combinations of any internalizing conditions with each cardiometabolic condition and any ICM-MM) on the number of haploinsufficient genes included in each deletion or the number of triplosensitive genes included in each duplication, adjusting for age, sex, Townsend deprivation index, the total number of genes included in each ND-CNV as a proxy of overall biological effect of each ND-CNV, and the first five genetic principal components to account for population stratification.

Interaction between CNVs and common genetic variation

To assess whether the association of common genetic variation and the risk of multimorbidity differs in individuals with and without an ND-CNV, the evidence for interaction between the presence of an ND-CNV and PRSs of our internalizing and cardiometabolic conditions of interest was assessed. PRSs for major depressive disorder (MDD),[40] anxiety,[41] low-density lipoprotein (LDL; as a proxy for dyslipidemia),[42] BMI (as a proxy of obesity),[43] systolic blood pressure (SBP; as a proxy for hypertension),[44] T2D,[45] and CKD[46] were computed. The genome-wide association studies (GWASs) used for the generation of these PRSs were selected because they were the latest and largest studies with publicly available summary statistics that excluded the UKBB. They are described in Table S1.

PRS-CS[47] was used for PRS calculation. PRS-CS is a Bayesian algorithm that can infer posterior effect sizes of SNPs via continuous shrinkage,[47] therefore avoiding the need for linkage disequilibrium pruning and p value thresholding. The inferred posterior effect sizes were used for PRS generation on PLINK 2.0.[48] In order to produce PRSs that are on the same scale across individuals from different ancestries, we adjusted them for ancestral differences in mean and variance using the 1000 Genomes dataset as a reference, as described by Khan et al.[49] The ancestry adjustment is described in detail elsewhere.[50]

Logistic regression analyses were performed as described previously, including the main effects of each ancestry-adjusted PRS, ND-CNV, and the interaction term PRS*ND-CNV.

Individual CNVs

We assessed the association of the combination of aggregated internalizing conditions with each cardiometabolic condition and any ICM-MM with each of the ND-CNVs individually. The number of individuals with each of the ND-CNVs is shown in Table S2. To ensure the analysis was statistically viable, CNVs that were observed fewer than 5 times in the total sample were excluded, resulting in 33 CNVs included in this analysis. The associations were assessed using logistic regression, adjusting for age at baseline, sex, Townsend deprivation index, and the first five genetic principal components.

Results

Associations of aggregated ND-CNVs with ICM-MM

There were 7,546 individuals (1.64%) with an ND-CNV in the UKBB. The demographic characteristics of the

Table 1. Counts and frequency for each of the ICM-MM phenotypes of interest in individuals with and without an ND-CNV

	ND-CNVs present	(N = 7,546)	ND-CNVs absent	(N = 451,937)
	Cases	Frequency (%)	Cases	Frequency (%)
Any internalizing and T2D	291	3.9	10,717	2.4
Any internalizing and obesity	397	5.3	15,580	3.5
Any internalizing and hypertension	764	10.1	37,688	8.3
Any internalizing and dyslipidemia	564	7.5	26,578	5.9
Any internalizing and CKD	193	2.6	8,085	1.8
Any ICM-MM	1,073	14.2	51,919	11.5

individuals with and without an ND-CNV are shown in Table S3. Individuals with a higher Townsend deprivation index were more likely to have an ND-CNV (odds ratio [OR] = 1.55, $p = 1.19 \times 10^{-34}$), meaning individuals with an ND-CNV were more likely to live in a more deprived environment than those without. The number of individuals with and without an ND-CNV who have a diagnosis of the eight conditions of interest is shown in Table S4.

14.2% of individuals with an ND-CNV had ICM-MM, compared to 11.5% of individuals without an ND-CNV. The number of individuals with and without an ND-CNV that have ICM-MM is shown in Table 1.

All combinations of ICM-MM were significantly associated with the presence of an ND-CNV after Bonferroni correction for multiple testing (Bonferroni p value threshold = 8.33×10^{-3}). The OR for any ICM-MM was similar to that for any internalizing condition or any cardiometabolic condition; however, the ORs for certain ICM-MM combinations (any internalizing condition and obesity, any internalizing condition and T2D, and any internalizing condition and CKD) were increased over any internalizing condition or any cardiometabolic condition separately. The associations between the presence of an ND-CNV and the different combinations of aggregated internalizing conditions with each cardiometabolic condition and ICM-MM are shown in Figure 1 and Table S5.

The associations remained significant when adjusting for BMI (Table S6). Restricting the analysis to individuals with both primary care and HES data (N = 229,951) did not substantively alter our findings (Figure S1; Table S7), while the ORs were higher and p values lower when including the full UKBB sample. Excluding individuals with proximal and distal deletions in 16p11.2 (N = 185), a known risk factor for early-onset morbid obesity, also did not substantively alter the findings (Figure S2; Table S8). When stratifying by ethnicity, the results for the White participants were similar to those of the total cohort, while no significant association was found for any other ethnicity (Table S9).

We examined whether the associations between having any ND-CNV and the phenotypic presentations of interest differed by sex. There was evidence of interaction between aggregated ND-CNVs and sex in association with any cardiometabolic condition and the combination of any internalizing condition and T2D (Figure 2; Table S10). Figure S3 shows that the increased risk of T2D or hypertension associated with an ND-CNV is higher in female than in male participants.

We examined whether the associations between the outcomes of interest differed for individuals with loss (deletion) or gain (duplication) of chromosomal material. Having a deletion increased the risk of having an internalizing condition and obesity over having a duplication (Figure 3; Table S11). When considering individual conditions, having a deletion was associated with greater odds of having obesity and T2D over having a duplication, while having a duplication was significantly associated with greater odds of having dyslipidemia over having a deletion, as seen in Figure S4.

The number of haploinsufficient genes within a deletion was significantly associated with all outcomes. The number of triplosensitive genes within a duplication did not show the same pattern (Figure S5). The total number of genes affected by a deletion or a duplication was significantly associated with all outcomes (Table S12).

Interaction between aggregated ND-CNVs and common genetic variation

The evidence for associations between ND-CNVs and combinations of ICM-MM remained significant when adjusting for each PRS (and vice versa). No interactions between PRS and aggregated ND-CNV were significant after Bonferroni correction for multiple testing. However, three interactions reached nominal significance ($p < 0.05$). These were the interaction of ND-CNV with anxiety PRS on the risk of any internalizing condition and obesity, along with any internalizing condition and CKD, and the interaction of ND-CNV with LDL PRS on the risk of any internalizing condition and CKD, as seen in Table S13.

Associations were also tested between the PRSs and ND-CNVs, as these could induce collider bias and therefore influence the ND-CNV*PRS interaction terms in the regression analyses. All ORs were very close to 1 for all PRSs, suggesting a lack of association and thus negligible

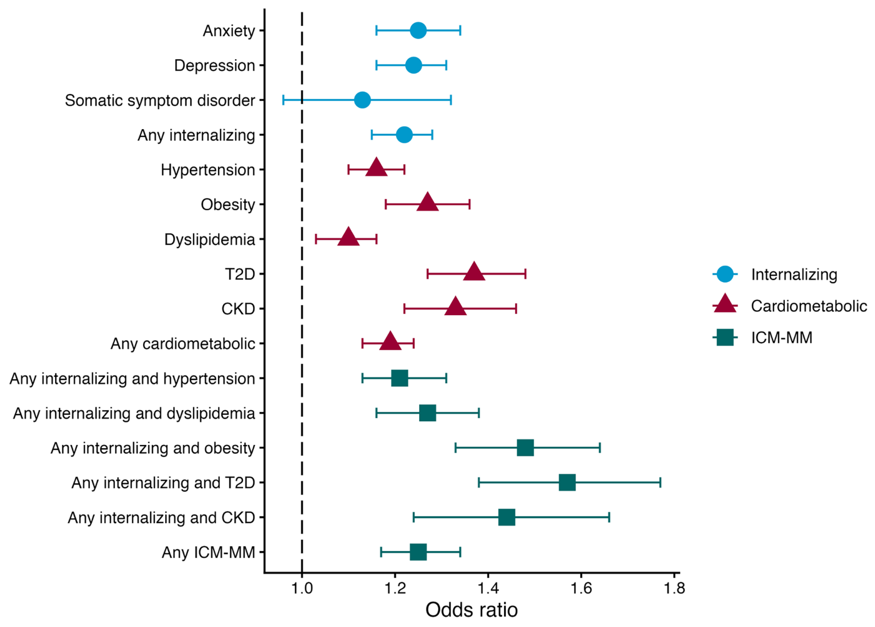

Figure 1. Association of ND-CNVs with individual conditions and multimorbidity
All outcomes are significantly associated with the presence of an ND-CNV after Bonferroni correction for multiple testing ($p < 8.33 \times 10^{-3}$), apart from somatic symptom disorder. The error bars indicate the 95% confidence interval of the odds ratio.

effects of collider bias, although the associations of MDD, anxiety, and CKD PRSs with ND-CNVs were nominally significant, due to the large sample size (Table S14).

Associations of individual ND-CNVs with ICM-MM

For each of the 33 ND-CNVs that were present more than 5 times (Table S1) in individuals in the UKBB, we investigated their association with the pairwise combinations of any internalizing condition with each cardiometabolic condition, as well as any ICM-MM (Figure S6; Table S15). After Bonferroni correction for multiple testing, 9 of the 33 NDD-CNVs showed evidence for association with ICM-MM ($p < 2.52 \times 10^{-4}$). Several deletions on the p arm of chromosome 16 were associated with multiple ICM-MM outcomes, while 22q11.2 duplication, 15q24 duplication, and 15q13.3 deletion were associated with any internalizing condition and obesity. All significant associations found indicated an increased risk of ICM-MM.

Discussion

The impact of ND-CNVs on the risk of multimorbidity between mental and physical health conditions has received little research attention to date. The aim of this study was to investigate the association of ND-CNVs with the presence of ICM-MM in a population cohort. Our findings indicate that individuals with ND-CNVs are more likely to experience ICM-MM, with particularly increased odds for specific combinations of internalizing and cardiometabolic conditions, such as any internalizing condition and T2D, any internalizing condition and CKD, and any internalizing condition and obesity. We also found evidence of differential associations with ICM-MM in individuals with ND-CNVs depending on sex and whether they carry a deletion or duplication and that genes intolerant to deletion (haploinsufficient genes) within ND-CNVs were more likely to contribute to ICM-MM risk than genes intolerant to duplication (triplosensitive genes). Finally, we found no evidence of interactions between ND-CNV status and PRSs of the internalizing and cardiometabolic conditions under study, indicating that common genetic variation does not modify the risk of ICM-MM in individuals with an ND-CNV.

We found that having any ND-CNV was associated with a 25% increased likelihood of ICM-MM, with the frequency of any ICM-MM in individuals with an ND-CNV being 14.2%, compared to 11.5% in individuals without an ND-CNV. Given the way UKBB participants were ascertained,[51] this is likely to be a lower bound estimate, as both the outcomes of interest and ND-CNVs are associated with premature mortality, and the older age of participants could introduce a survival bias. While ND-CNVs have mostly been studied in relation to NDCs[52–54] and other psychiatric conditions,[25,26,55] they have also recently been linked to multiple physical health outcomes,[27,56] including obesity,[36,57] renal disease,[58] and diabetes.[59] While much of the literature linking ND-CNVs with physical health outcomes has focused on clinical populations and rare and severe syndromic phenotypes, we studied a population cohort that is not enriched for more severe manifestations of ND-CNVs. While unknown, it is also likely that many of the individuals with ND-CNVs in the UKBB are unaware of their genotype. We explored common, earlier-onset cardiometabolic conditions that are on the causal pathway to cardiovascular morbidity and mortality, and we found that ND-CNVs were significantly associated with all the cardiometabolic conditions we examined, as well as ICM-MM. Our results suggest that ND-CNVs are associated with common conditions and ICM-MM even in the general population, while it is important to keep in mind that these outcomes are likely to be more severe in clinically ascertained carriers. Associations between ND-CNVs and ICM-MM remained significant when removing ND-CNVs that are known risk factors for morbid obesity or when correcting

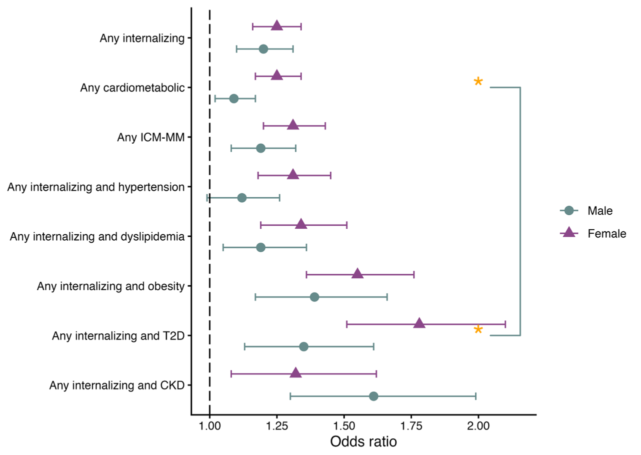

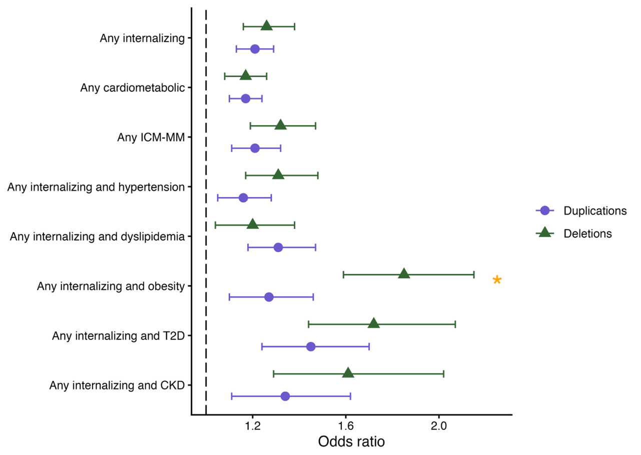

Figure 2. Association of ND-CNVs with multimorbidity for male and female sex
Stars indicate a significant interaction between the presence of ND-CNVs and sex ($p < 0.05$). The error bars indicate the 95% confidence interval of the odds ratio.

Figure 3. Association of duplications and deletions with multimorbidity
Stars indicate a suggested difference between deletions and duplications ($p < 0.05$). The error bars indicate the 95% confidence interval of the odds ratio.

for BMI, suggesting that in this dataset, the associations are unlikely to be driven by the effect of ND-CNVs on body mass.

In addition to our composite phenotype of any ICM-MM, we also assessed the impact of ND-CNVs on pairwise combinations of any internalizing condition and each of the five cardiometabolic conditions. Our rationale was that while the internalizing disorders are highly phenotypically and genetically correlated,[50,60,61] the cardiometabolic disorders are more heterogeneous; therefore, the associations with ND-CNVs may differ between the pairwise ICM-MM combinations. Our findings confirm this assumption. While the odds for any ICM-MM conferred by ND-CNVs were quite similar to those for any internalizing condition or any cardiometabolic condition alone, the odds for some of the combinations (e.g., any internalizing condition and obesity, any internalizing condition and T2D, and any internalizing condition and CKD) were higher than those for any internalizing condition, any cardiometabolic condition, or any ICM-MM. This indicates that individuals with ND-CNVs have higher odds of developing certain combinations of internalizing and cardiometabolic conditions than others and could benefit from closer monitoring for early indications of particular conditions.

Interestingly, we observed that the effect of the presence of an ND-CNV on the odds of any cardiometabolic condition, as well as any internalizing condition and T2D, appeared to be higher in female than in male participants. While there was no strong evidence for the remaining outcomes, there was a pattern for larger effects for female than for male participants for all ICM-MM combinations apart from any internalizing condition and CKD. Previous studies on sex differences in the effect of ND-CNVs on NDCs and other psychiatric disorders have produced conflicting results,[26,62–65] with some studies reporting higher rates of depression in female adults with ND-CNVs[26] and of anxiety and depression in female children with a diagnosed NDC and an ND-CNV,[65] while others found no evidence of sex differences in ND-CNV burden for anxiety and depression.[62] While further studies are required to understand the underlying mechanisms behind possible sex differences, our findings highlight the importance of considering sex as a factor when designing tailored health monitoring strategies for individuals with ND-CNVs.

Moreover, we found suggestive evidence of differences between the effects of deletions and duplications on ICM-MM, with deletions being associated with higher odds of having any internalizing condition and obesity compared with duplications. Deletions in the 16p11.2 region are known risk factors for class III obesity,[36–38] and it is likely that these regions contribute to this finding; however, the association between ND-CNVs and any internalizing condition and obesity remained significant when deletions in this region were removed, suggesting that other ND-CNVs also predispose to this type of multimorbidity. In order to further explore the way in which ND-CNVs are involved in the pathogenesis of multimorbidity, we examined the association of dosage-sensitive genes within an ND-CNV with ICM-MM. Haploinsufficient genes (those intolerant to deletion) within ND-CNVs were associated with higher odds of most combinations of ICM-MM, whereas triplosensitive genes (those intolerant to duplication) did not show evidence of a strong effect. The total number of genes within both deletions and duplications was significantly associated with most combinations of ICM-MM; however, the effect size was small. Our findings suggest that, for the phenotypes we studied, in individuals with deletions, the loss of haploinsufficient genes is pathogenic; however, the same might not be

the case for duplications, where the total number of genes hit or other parameters, such as the total length or the penetrance of the variant, might be more important than the number of triplosensitive genes duplicated. Importantly, for individuals with deletions, the number of haploinsufficient genes can be used to identify individuals who might be at risk of developing a more severe phenotype.

We explored the possibility of interaction between the presence of an ND-CNV and PRSs of our internalizing and cardiometabolic traits of interest on the risk of ICM-MM. The absence of detectable interactions between ND-CNVs and the PRSs suggests that the effect of ND-CNVs on the risk of ICM-MM is not modified by the effect of common genetic variation. Although the UKBB is one of the largest cohorts internationally available for the study of our aims, ND-CNVs are rare (they were present in 1.6% of participants), and it is likely that we lacked sufficient statistical power to detect possible interactions; however, our results are in agreement with previous studies exploring the effect of CNVs or other rare variants and common variation on depression phenotypes in cohorts of similar or smaller size.[66–68]

When examining the ND-CNVs individually, we found nine with evidence for association with at least one type of ICM-MM. Four of these were deletions on the p arm of chromosome 16 (16p13.11 deletion, 16p11.2 deletion, 16p12.1 deletion, and 16p12.1 distal deletion). 16p11.2 deletion, 16p12.1 deletion, and 16p13.11 deletion were associated with all combinations of ICM-MM, while 16p12.1 distal deletion was associated with any internalizing condition and T2D and any internalizing condition and obesity. These results are consistent with previous research that found that deletions in this region are associated with psychiatric disorders and traits,[25,52] obesity,[36–38] T2D,[37] and kidney disease biomarkers.[37] 22q11.2 duplication was associated with any internalizing condition and obesity. This variant is considered to be associated with a variable and mild phenotype compared to deletions in the same region.[69] Our results highlight the considerable heterogeneity between ND-CNVs. Where increasing sizes of population-based cohorts allow the opportunity, each CNV should be studied in isolation to better understand its specific contribution to disease risk and provide valuable insight into the biological underpinnings of ICM-MM.

Our findings give evidence of an association between ND-CNVs and the co-occurrence of common internalizing and cardiometabolic conditions, a multimorbid presentation that can lead to substantive functional impairment, a reduced quality of life, and premature mortality. This finding has several clinical implications. Firstly, it underscores the need for healthcare systems to adopt a more holistic approach to multimorbidity, particularly in relation to individuals with rare genetic variants. Current healthcare service provision tends to look at each diagnosis in isolation, but it is evident from our findings that this approach may not be suited for individuals with ND-CNVs, who are at risk of multiple conditions throughout their lifetime, many of which need to be managed by different medical specialties. It is imperative that healthcare systems transition to a more comprehensive multidisciplinary model of care, particularly for individuals with known pathogenic genetic variants. Secondly, our results suggest that the impacts of ND-CNVs extend beyond the phenotypes they are traditionally associated with (e.g., congenital abnormalities, intellectual disability, autism, and schizophrenia) to include common morbidities and multimorbidity seen in older-age people in the general population. Recent breakthroughs in sequencing technology allow for the identification of an increasing range of rare genetic variants by medical genomics services, and children with NDCs will often be screened for ND-CNVs. ND-CNVs are often inherited, and reduced penetrance may mean that seemingly unaffected relatives might carry an ND-CNV that predisposes them to ICM-MM and could also stand to benefit from screening.

There are some limitations to our study. The UKBB is one of the largest publicly available population cohorts and was designed as a prospective study of middle and older age,[31] with the median age at recruitment being 57 years. It is therefore well suited to this study, as the age range of the participants allowed us to study conditions that tend to appear later in life. However, it is also susceptible to survival bias. As multimorbidity and CNV burden are associated with premature mortality,[56,70] it is likely that individuals with the most severe outcomes are underrepresented, which would lead to an effective reduction of multimorbidity frequency and effect sizes compared to the general population. Moreover, the participants of the UKBB have been found to have higher socioeconomic status, report higher overall health, and be less ethnically diverse than the average UK population,[71] which could also lead to reduced population-based effect sizes. Further studies are required to assess the generalizability of our findings to populations with different characteristics from the UKBB, particularly to individuals of non-European ancestry and low socioeconomic status. When assessing the interaction between ND-CNVs and PRSs, we found that some of the PRSs were associated with ND-CNV status (MDD, anxiety, and CKD PRSs). These associations could introduce a collider bias in the analysis, whereby adjusting for PRS would alter the ND-CNV association with the outcomes in a manner that resembles an interaction. However, we did not find evidence of interaction between ND-CNVs and any of the PRSs in our analysis. Finally, the variants we examined tend to be low in frequency, some appearing in fewer than 100 instances in half a million UKBB participants. We aggregated ND-CNVs to have increased statistical power, which facilitated discovering associations

that would otherwise be missed. This approach provides valuable insights into the common effects of the variants but does not allow for the clarification of ND-CNV-specific relationships.

In conclusion, this study demonstrates that, on a population level, individuals with ND-CNVs have higher odds of developing common and preventable forms of ICM-MM. As the field of medical genetics continues to expand, understanding how rare genetic variants contribute to multimorbidity will be essential for improving the health outcomes of affected individuals.

Data and code availability

The code associated with all analyses described can be found at https://github.com/Zan-K/icm-mm_cnv_analysis.

Consortia

The members of the LINC consortium are Marianne B.M. van den Bree, George Kirov, Michael J. Owen, James T.R. Walters, Peter A. Holmans, Jane Lynch, Ioanna K. Katzourou, Jack F.J. Underwood, David A. van Heel, Sarah Finer, Daniel Stow, Golam M. Khandaker, Nicholas J. Timpson, John A. A. MacLeod, Julie P. Clayton, Ruby S.M. Tsang, Jane Sprackman, Shahid Khan, Inês Barroso, Rupert A. Payne, Mark Mon-Williams, Megan L. Wood, Nabila Ali, Hilary C. Martin, Thomas Werge, Andrés Ingason, Morteza Vaez, and Lam O. Huang.

Acknowledgments

This research has been conducted using the UK Biobank Resource under application number 79704. This work was funded by the Tackling Multimorbidity at Scale Strategic Priorities Fund Programme (MR/W014416/1) delivered by the Medical Research Council and the National Institute for Health Research in partnership with the Economic and Social Research Council and in collaboration with the Engineering and Physical Sciences Research Council. I.K.K. is supported by this grant. We thank the members of the LINC study public advisory group for their contribution.

Author contributions

Study conceptualization and design, I.K.K., M.B.M.v.d.B., P.H., G.K., M.J.O., J.W., and LINC consortium members; analytical consultation and interpretation, I.K.K., M.B.M.v.d.B., P.H., G.K., M.J.O., J.W., R.T., D.S., and I.B.; UKBB data curation, I.K.K.; genetic data preparation, I.K.K.; supervision, M.B.M.v.d.B., P.H., G.K., M.J.O., P.H., and J.W. All listed authors critically edited the manuscript.

Declaration of interests

M.J.O. receives research grants from Takeda Pharmaceuticals and Akrivia Health.

Supplemental information

Supplemental information can be found online at https://doi.org/10.1016/j.ajhg.2026.02.021.

Received: May 15, 2025
Accepted: February 26, 2026
Published: April 6, 2026

References

1. The Academy of Medical Sciences. Multimorbidity: A Priority for Global Health Research. [Internet]. 2018. Available from: https://acmedsci.ac.uk/file-download/82222577
2. World Health Organization (2016). Multimorbidity [Internet]. Geneva (World Health Organization). [cited 2025 Jan 16]. 28 p. (Technical Series on Safer Primary Care). Available from:. https://iris.who.int/handle/10665/252275.
3. Barnett, K., Mercer, S.W., Norbury, M., Watt, G., Wyke, S., and Guthrie, B. (2012). Epidemiology of multimorbidity and implications for health care, research, and medical education: a cross-sectional study. Lancet Lond Engl *380*, 37–43.
4. Whiteford, H.A., Degenhardt, L., Rehm, J., Baxter, A.J., Ferrari, A.J., Erskine, H.E., Charlson, F.J., Norman, R.E., Flaxman, A.D., Johns, N., et al. (2013). Global burden of disease attributable to mental and substance use disorders: findings from the Global Burden of Disease Study 2010. Lancet Lond Engl *382*, 1575–1586.
5. GBD 2013 DALYs and HALE Collaborators, Murray, C.J.L., Barber, R.M., Foreman, K.J., Abbasoglu Ozgoren, A., Abd-Allah, F., Abera, S.F., Aboyans, V., Abraham, J.P., Abubakar, I., et al. (2015). Global, regional, and national disability-adjusted life years (DALYs) for 306 diseases and injuries and healthy life expectancy (HALE) for 188 countries, 1990-2013: quantifying the epidemiological transition. Lancet Lond Engl *386*, 2145–2191.
6. Mezuk, B., Eaton, W.W., Albrecht, S., and Golden, S.H. (2008). Depression and type 2 diabetes over the lifespan: a meta-analysis. Diabetes Care *31*, 2383–2390.
7. Cohen, B.E., Edmondson, D., and Kronish, I.M. (2015). State of the Art Review: Depression, Stress, Anxiety, and Cardiovascular Disease. Am. J. Hypertens. *28*, 1295–1302.
8. Yu, M., Zhang, X., Lu, F., and Fang, L. (2015). Depression and Risk for Diabetes: A Meta-Analysis. Can. J. Diabetes *39*, 266–272.
9. Nicholson, A., Kuper, H., and Hemingway, H. (2006). Depression as an aetiologic and prognostic factor in coronary heart disease: a meta-analysis of 6362 events among 146 538 participants in 54 observational studies. Eur. Heart J. *27*, 2763–2774.
10. Dickens, C. (2015). Depression in people with coronary heart disease: prognostic significance and mechanisms. Curr. Cardiol. Rep. *17*, 83.
11. Anderson, R.J., Freedland, K.E., Clouse, R.E., and Lustman, P.J. (2001). The prevalence of comorbid depression in adults with diabetes: a meta-analysis. Diabetes Care *24*, 1069–1078.
12. Fulton, S., Décarie-Spain, L., Fioramonti, X., Guiard, B., and Nakajima, S. (2022). The menace of obesity to depression and anxiety prevalence. Trends Endocrinol Metab TEM *33*, 18–35.
13. Laursen, T.M., Musliner, K.L., Benros, M.E., Vestergaard, M., and Munk-Olsen, T. (2016). Mortality and life expectancy in

persons with severe unipolar depression. J. Affect. Disord. *193*, 203–207.
14. May, H.T., Horne, B.D., Knight, S., Knowlton, K.U., Bair, T.L., Lappé, D.L., Le, V.T., and Muhlestein, J.B. (2017). The association of depression at any time to the risk of death following coronary artery disease diagnosis. Eur. Heart J. Qual. Care Clin. Outcomes *3*, 296–302.
15. Dong, G., Feng, J., Sun, F., Chen, J., and Zhao, X.M. (2021). A global overview of genetically interpretable multimorbidities among common diseases in the UK Biobank. Genome Med. *13*, 110.
16. Amare, A.T., Schubert, K.O., Klingler-Hoffmann, M., Cohen-Woods, S., and Baune, B.T. (2017). The genetic overlap between mood disorders and cardiometabolic diseases: a systematic review of genome wide and candidate gene studies. Transl. Psychiatry *7*, e1007.
17. Wong, B.C.F., Chau, C.K.L., Ao, F.K., Mo, C.H., Wong, S.Y., Wong, Y.H., and So, H.C. (2019). Differential associations of depression-related phenotypes with cardiometabolic risks: Polygenic analyses and exploring shared genetic variants and pathways. Depress. Anxiety *36*, 330–344.
18. Hagenaars, S.P., Coleman, J.R.I., Choi, S.W., Gaspar, H., Adams, M.J., Howard, D.M., Hodgson, K., Traylor, M., Air, T.M., Andlauer, T.F.M., et al. (2020). Genetic comorbidity between major depression and cardio-metabolic traits, stratified by age at onset of major depression. Am. J. Med. Genet. B Neuropsychiatr. Genet. *183*, 309–330.
19. Baranova, A., Liu, D., Chandhoke, V., Cao, H., and Zhang, F. (2025). Unraveling the genetic links between depression and type 2 diabetes. Prog. Neuropsychopharmacol. Biol. Psychiatry *137*, 111258.
20. Lee, C., and Scherer, S.W. (2010). The clinical context of copy number variation in the human genome. Expert Rev. Mol. Med. *12*, e8.
21. Coe, B.P., Witherspoon, K., Rosenfeld, J.A., van Bon, B.W.M., Vulto-van Silfhout, A.T., Bosco, P., Friend, K.L., Baker, C., Buono, S., Vissers, L.E.L.M., et al. (2014). Refining analyses of copy number variation identifies specific genes associated with developmental delay. Nat. Genet. *46*, 1063–1071.
22. Cooper, G.M., Coe, B.P., Girirajan, S., Rosenfeld, J.A., Vu, T.H., Baker, C., Williams, C., Stalker, H., Hamid, R., Hannig, V., et al. (2011). A copy number variation morbidity map of developmental delay. Nat. Genet. *43*, 838–846.
23. Rees, E., Walters, J.T.R., Georgieva, L., Isles, A.R., Chambert, K.D., Richards, A.L., Mahoney-Davies, G., Legge, S.E., Moran, J.L., McCarroll, S.A., et al. (2014). Analysis of copy number variations at 15 schizophrenia-associated loci. Br. J. Psychiatry *204*, 108–114.
24. Truty, R., Paul, J., Kennemer, M., Lincoln, S.E., Olivares, E., Nussbaum, R.L., and Aradhya, S. (2019). Prevalence and properties of intragenic copy-number variation in Mendelian disease genes. Genet. Med. *21*, 114–123.
25. Chawner, S.J.R.A., Owen, M.J., Holmans, P., Raymond, F.L., Skuse, D., Hall, J., and van den Bree, M.B.M. (2019). Genotype–phenotype associations in children with copy number variants associated with high neuropsychiatric risk in the UK (IMAGINE-ID): a case-control cohort study. Lancet Psychiatry *6*, 493–505.
26. Kendall, K.M., Rees, E., Bracher-Smith, M., Legge, S., Riglin, L., Zammit, S., O'Donovan, M.C., Owen, M.J., Jones, I., Kirov, G., and Walters, J.T.R. (2019). Association of Rare Copy Number Variants With Risk of Depression. JAMA Psychiatry *76*, 818–825.
27. Crawford, K., Bracher-Smith, M., Owen, D., Kendall, K.M., Rees, E., Pardiñas, A.F., Einon, M., Escott-Price, V., Walters, J.T.R., O'Donovan, M.C., et al. (2019). Medical consequences of pathogenic CNVs in adults: analysis of the UK Biobank. J. Med. Genet. *56*, 131–138.
28. Voll, S.L., Boot, E., Butcher, N.J., Cooper, S., Heung, T., Chow, E.W.C., Silversides, C.K., and Bassett, A.S. (2017). Obesity in adults with 22q11.2 deletion syndrome. Genet. Med. *19*, 204–208.
29. Van, L., Heung, T., Malecki, S.L., Fenn, C., Tyrer, A., Sanches, M., Chow, E.W.C., Boot, E., Corral, M., Dash, S., et al. (2020). 22q11.2 microdeletion and increased risk for type 2 diabetes. eClinicalMedicine *26*, 100528.
30. Perrone, L., Marzuillo, P., Grandone, A., and del Giudice, E.M. (2010). Chromosome 16p11.2 deletions: another piece in the genetic puzzle of childhood obesity. Ital. J. Pediatr. *36*, 43.
31. Bycroft, C., Freeman, C., Petkova, D., Band, G., Elliott, L.T., Sharp, K., Motyer, A., Vukcevic, D., Delaneau, O., O'Connell, J., et al. (2018). The UK Biobank resource with deep phenotyping and genomic data. Nature *562*, 203–209.
32. Kendall, K.M., Rees, E., Escott-Price, V., Einon, M., Thomas, R., Hewitt, J., O'Donovan, M.C., Owen, M.J., Walters, J.T.R., and Kirov, G. (2017). Cognitive Performance Among Carriers of Pathogenic Copy Number Variants: Analysis of 152,000 UK Biobank Subjects. Biol. Psychiatry *82*, 103–110.
33. Wang, K., Li, M., Hadley, D., Liu, R., Glessner, J., Grant, S.F.A., Hakonarson, H., and Bucan, M. (2007). PennCNV: An integrated hidden Markov model designed for high-resolution copy number variation detection in whole-genome SNP genotyping data. Genome Res. *17*, 1665–1674.
34. R Core Team (2022). R: A Language and Environment for Statistical Computing (Vienna, Austria: R Foundation for Statistical Computing). https://www.R-project.org/.
35. FIRTH, D. (1993). Bias reduction of maximum likelihood estimates. Biometrika *80*, 27–38.
36. Bochukova, E.G., Huang, N., Keogh, J., Henning, E., Purmann, C., Blaszczyk, K., Saeed, S., Hamilton-Shield, J., Clayton-Smith, J., O'Rahilly, S., et al. (2010). Large, rare chromosomal deletions associated with severe early-onset obesity. Nature *463*, 666–670.
37. Hanssen, R., Auwerx, C., Jõeloo, M., Sadler, M.C., Estonian Biobank Research Team, Henning, E., Keogh, J., Bounds, R., Smith, M., Firth, H.V., et al. (2023). Chromosomal deletions on 16p11.2 encompassing *SH2B1* are associated with accelerated metabolic disease. Cell Rep. Med. *4*, 101155.
38. Walters, R.G., Jacquemont, S., Valsesia, A., de Smith, A.J., Martinet, D., Andersson, J., Falchi, M., Chen, F., Andrieux, J., Lobbens, S., et al. (2010). A new highly penetrant form of obesity due to deletions on chromosome 16p11.2. Nature *463*, 671–675.
39. Collins, R.L., Glessner, J.T., Porcu, E., Lepamets, M., Brandon, R., Lauricella, C., Han, L., Morley, T., Niestroj, L.M., Ulirsch, J., et al. (2022). A cross-disorder dosage sensitivity map of the human genome. Cell *185*, 3041–3055.e25. https://doi.org/10.1016/j.cell.2022.06.036.
40. Wray, N.R., Ripke, S., Mattheisen, M., Trzaskowski, M., Byrne, E.M., Abdellaoui, A., Adams, M.J., Agerbo, E., Air, T.M., Andlauer, T.M.F., et al. (2018). Genome-wide association

analyses identify 44 risk variants and refine the genetic architecture of major depression. Nat. Genet. *50*, 668–681.
41. Meier, S.M., Trontti, K., Purves, K.L., Als, T.D., Grove, J., Laine, M., Pedersen, M.G., Bybjerg-Grauholm, J., Bækved-Hansen, M., Sokolowska, E., et al. (2019). Genetic Variants Associated With Anxiety and Stress-Related Disorders: A Genome-Wide Association Study and Mouse-Model Study. JAMA Psychiatry *76*, 924–932.
42. Willer, C.J., Schmidt, E.M., Sengupta, S., Peloso, G.M., Gustafsson, S., Kanoni, S., Ganna, A., Chen, J., Buchkovich, M.L., Mora, S., et al. (2013). Discovery and Refinement of Loci Associated with Lipid Levels. Nat. Genet. *45*, 1274–1283.
43. Locke, A.E., Kahali, B., Berndt, S.I., Justice, A.E., Pers, T.H., Day, F.R., Powell, C., Vedantam, S., Buchkovich, M.L., Yang, J., et al. (2015). Genetic studies of body mass index yield new insights for obesity biology. Nature *518*, 197–206.
44. Keaton, J.M., Kamali, Z., Xie, T., Vaez, A., Williams, A., Goleva, S.B., Ani, A., Evangelou, E., Hellwege, J.N., Yengo, L., et al. (2024). Genome-wide analysis in over 1 million individuals of European ancestry yields improved polygenic risk scores for blood pressure traits. Nat. Genet. *56*, 778–791.
45. Mahajan, A., Taliun, D., Thurner, M., Robertson, N.R., Torres, J.M., Rayner, N.W., Payne, A.J., Steinthorsdottir, V., Scott, R.A., Grarup, N., et al. (2018). Fine-mapping type 2 diabetes loci to single-variant resolution using high-density imputation and islet-specific epigenome maps. Nat. Genet. *50*, 1505–1513. https://doi.org/10.1038/s41588-018-0241-6.
46. Pattaro, C., Teumer, A., Gorski, M., Chu, A.Y., Li, M., Mijatovic, V., Garnaas, M., Tin, A., Sorice, R., Li, Y., et al. (2016). Genetic associations at 53 loci highlight cell types and biological pathways relevant for kidney function. Nat. Commun. *7*, 10023. https://doi.org/10.1038/ncomms10023.
47. Ge, T., Chen, C.Y., Ni, Y., Feng, Y.C.A., and Smoller, J.W. (2019). Polygenic prediction via Bayesian regression and continuous shrinkage priors. Nat. Commun. *10*, 1776.
48. Purcell, S., Neale, B., Todd-Brown, K., Thomas, L., Ferreira, M.A.R., Bender, D., Maller, J., Sklar, P., de Bakker, P.I.W., Daly, M.J., and Sham, P.C. (2007). PLINK: A Tool Set for Whole-Genome Association and Population-Based Linkage Analyses. Am. J. Hum. Genet. *81*, 559–575.
49. Khan, A., Turchin, M.C., Patki, A., Srinivasasainagendra, V., Shang, N., Nadukuru, R., Jones, A.C., Malolepsza, E., Dikilitas, O., Kullo, I.J., et al. (2022). Genome-wide polygenic score to predict chronic kidney disease across ancestries. Nat. Med. *28*, 1412–1420.
50. Katzourou, I.K., LINC Consortium, Barroso, I., Benger, L., Ingason, A., Stow, D., Tsang, R., Wood, M., Kirov, G., Walters, J., et al. (2025). Contributions of common and rare genetic variation to different measures of mood and anxiety disorder in the UK Biobank. BJPsych Open *11*, e97.
51. van Alten, S., Domingue, B.W., Faul, J., Galama, T., and Marees, A.T. (2024). Reweighting UK Biobank corrects for pervasive selection bias due to volunteering. Int. J. Epidemiol. *53*, dyae054.
52. Niarchou, M., Chawner, S.J.R.A., Doherty, J.L., Maillard, A.M., Jacquemont, S., Chung, W.K., Green-Snyder, L., Bernier, R.A., Goin-Kochel, R.P., Hanson, E., et al. (2019). Psychiatric disorders in children with 16p11.2 deletion and duplication. Transl. Psychiatry *9*, 8.
53. Linden, S.C., Watson, C.J., Smith, J., Chawner, S.J.R.A., Lancaster, T.M., Evans, F., Williams, N., Skuse, D., Raymond, F.L., Hall, J., et al. (2021). The psychiatric phenotypes of 1q21 distal deletion and duplication. Transl. Psychiatry *11*, 105.
54. Schneider, M., Debbané, M., Bassett, A.S., Chow, E.W.C., Fung, W.L.A., van den Bree, M., Owen, M., Murphy, K.C., Niarchou, M., Kates, W.R., et al. (2014). Psychiatric Disorders From Childhood to Adulthood in 22q11.2 Deletion Syndrome: Results From the International Consortium on Brain and Behavior in 22q11.2 Deletion Syndrome. Am. J. Psychiatry *171*, 627–639.
55. Adams, R.L., Baird, A., Smith, J., Williams, N., van den Bree, M.B.M., Linden, D.E.J., Owen, M.J., Hall, J., and Linden, S.C. (2023). Psychopathology in adults with copy number variants. Psychol. Med. *53*, 3142–3149.
56. Auwerx, C., Lepamets, M., Sadler, M.C., Patxot, M., Stojanov, M., Baud, D., Mägi, R., Estonian Biobank Research Team, Porcu, E., Reymond, A., and Kutalik, Z. (2022). The individual and global impact of copy-number variants on complex human traits. Am. J. Hum. Genet. *109*, 647–668.
57. Stahel, P., Nahmias, A., Sud, S.K., Lee, S.J., Pucci, A., Yousseif, A., Youseff, A., Jackson, T., Urbach, D.R., Okrainec, A., et al. (2019). Evaluation of the Genetic Association Between Adult Obesity and Neuropsychiatric Disease. Diabetes *68*, 2235–2246.
58. Wu, C.H.W., Lim, T.Y., Wang, C., Seltzsam, S., Zheng, B., Schierbaum, L., Schneider, S., Mann, N., Connaughton, D.M., Nakayama, M., et al. (2022). Copy Number Variation Analysis Facilitates Identification of Genetic Causation in Patients with Congenital Anomalies of the Kidney and Urinary Tract. Eur. Urol. Open Sci. *44*, 106–112. https://doi.org/10.1016/j.euros.2022.08.004.
59. Berberich, A.J., Huot, C., Cao, H., McIntyre, A.D., Robinson, J.F., Wang, J., and Hegele, R.A. (2019). Copy Number Variation in GCK in Patients With Maturity-Onset Diabetes of the Young. J. Clin. Endocrinol. Metab. *104*, 3428–3436.
60. Mei, L., Gao, Y., Chen, M., Zhang, X., Yue, W., Zhang, D., and Yu, H. (2022). Overlapping common genetic architecture between major depressive disorders and anxiety and stress-related disorders. Prog. Neuropsychopharmacol. Biol. Psychiatry *113*, 110450.
61. Kessler, R.C., Gruber, M., Hettema, J.M., Hwang, I., Sampson, N., and Yonkers, K.A. (2008). Co-morbid major depression and generalized anxiety disorders in the National Comorbidity Survey follow-up. Psychol. Med. *38*, 365–374.
62. Martin, J., Asjadi, K., Hubbard, L., Kendall, K., Pardiñas, A.F., Jermy, B., Lewis, C.M., Baune, B.T., Boomsma, D.I., Hamilton, S.P., et al. (2021). Examining sex differences in neurodevelopmental and psychiatric genetic risk in anxiety and depression. PLoS One *16*, e0248254.
63. Polyak, A., Rosenfeld, J.A., and Girirajan, S. (2015). An assessment of sex bias in neurodevelopmental disorders. Genome Med. *7*, 94.
64. Jung, B., Ahn, K., Justice, C., Norman, L., Price, J., Sudre, G., and Shaw, P. (2023). Rare copy number variants in males and females with childhood attention-deficit/hyperactivity disorder. Mol. Psychiatry *28*, 1240–1247.
65. Martin, J., Tammimies, K., Karlsson, R., Lu, Y., Larsson, H., Lichtenstein, P., and Magnusson, P.K.E. (2019). Copy number variation and neuropsychiatric problems in females and males in the general population. Am. J. Med. Genet. *180*, 341–350.
66. Mollon, J., Schultz, L.M., Huguet, G., Knowles, E.E.M., Mathias, S.R., Rodrigue, A., Alexander-Bloch, A., Saci, Z.,

Jean-Louis, M., Kumar, K., et al. (2023). Impact of Copy Number Variants and Polygenic Risk Scores on Psychopathology in the UK Biobank. Biol. Psychiatry *94*, 591–600.

67. Vaez, M., Montalbano, S., Waples, R., Krebs, M.D., Hellberg, K.L.G., Gådin, J., Bybjerg-Grauholm, J., Mortensen, P.B., Børglum, A.D., Nordentoft, M., et al. (2024). Evaluating the Joint Effects of Recurrent Copy Number Variants and Polygenic Scores on the Risk of Psychiatric Disorders in the iPSYCH2015 Case-Cohort Sample. medRxiv. https://doi.org/10.1101/2024.09.23.24314234.

68. Tian, R., Ge, T., Kweon, H., Rocha, D.B., Lam, M., Liu, J.Z., Singh, K., Biogen Biobank Team, Levey, D.F., Gelernter, J., et al. (2024). Whole-exome sequencing in UK Biobank reveals rare genetic architecture for depression. Nat. Commun. *15*, 1755.

69. Zamariolli, M., Auwerx, C., Sadler, M.C., van der Graaf, A., Lepik, K., Schoeler, T., Moysés-Oliveira, M., Dantas, A.G., Melaragno, M.I., and Kutalik, Z. (2023). The impact of 22q11.2 copy-number variants on human traits in the general population. Am. J. Hum. Genet. *110*, 300–313.

70. Chowdhury, S.R., Chandra Das, D., Sunna, T.C., Beyene, J., and Hossain, A. (2023). Global and regional prevalence of multimorbidity in the adult population in community settings: a systematic review and meta-analysis. eClinicalMedicine *57*, 101860. https://doi.org/10.1016/j.eclinm.2023.101860.

71. Fry, A., Littlejohns, T.J., Sudlow, C., Doherty, N., Adamska, L., Sprosen, T., Collins, R., and Allen, N.E. (2017). Comparison of Sociodemographic and Health-Related Characteristics of UK Biobank Participants With Those of the General Population. Am. J. Epidemiol. *186*, 1026–1034.

The American Journal of Human Genetics, Volume *113*

Supplemental information

Neurodevelopmental copy-number variants increase risk of internalizing and cardiometabolic multimorbidity: Findings from the UK Biobank

Ioanna K. Katzourou, LINC consortium, Inês Barroso, Julie Clayton, Golam Khandaker, Daniel Stow, Nicolas Timpson, Ruby Tsang, Jack Underwood, Megan Wood, George Kirov, James Walters, Michael J. Owen, Peter Holmans, and Marianne B. M. van den Bree

SUPPLEMENTAL NOTES

The lists of clinical codes curated by the MULTIPLY project (https://zenodo.org/record/7643566) were used to establish instances of the conditions of interest(1). The primary care codelists for depression and anxiety were amended by psychiatrists within the LINC team. Dyslipidemia is not defined within the MULTIPLY clinical code lists, therefore the clinical code list from Baksh *et al*. was used to identify instances of dyslipidemia within primary care records(2), and ICD-10 codes E78.0, E78.1, E78.2, E78.3, E78.4, E78.5, E88.81 were used to define dyslipidemia in HES.

SUPPLEMENTAL FIGURES

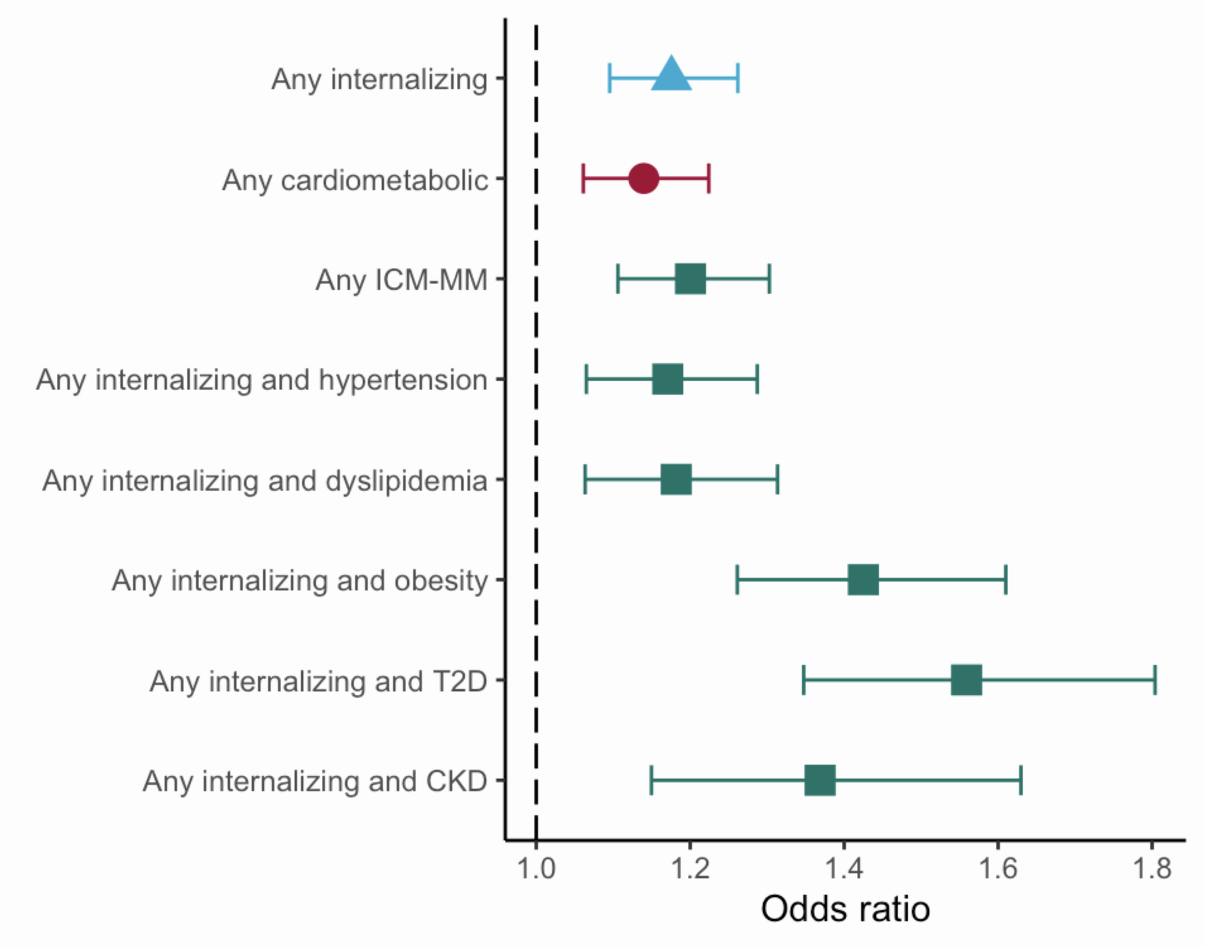

Figure S1. Association of ND-CNVs with multimorbidity for individuals with both primary care and HES data (N= 229,951).

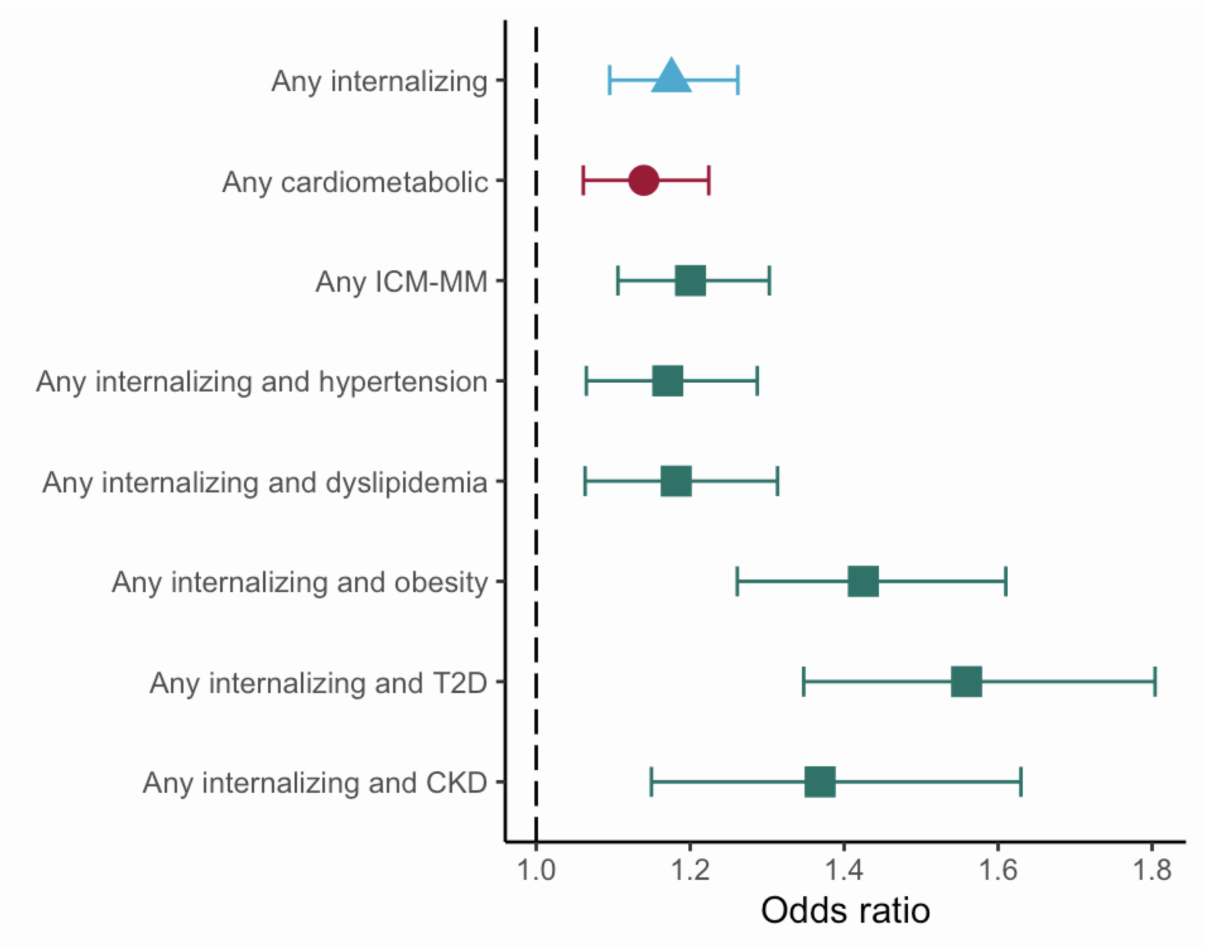

Figure S2. Association of ND-CNVs with multimorbidity excluding individuals with 16p11.2 proximal and distal deletions (N= 185).

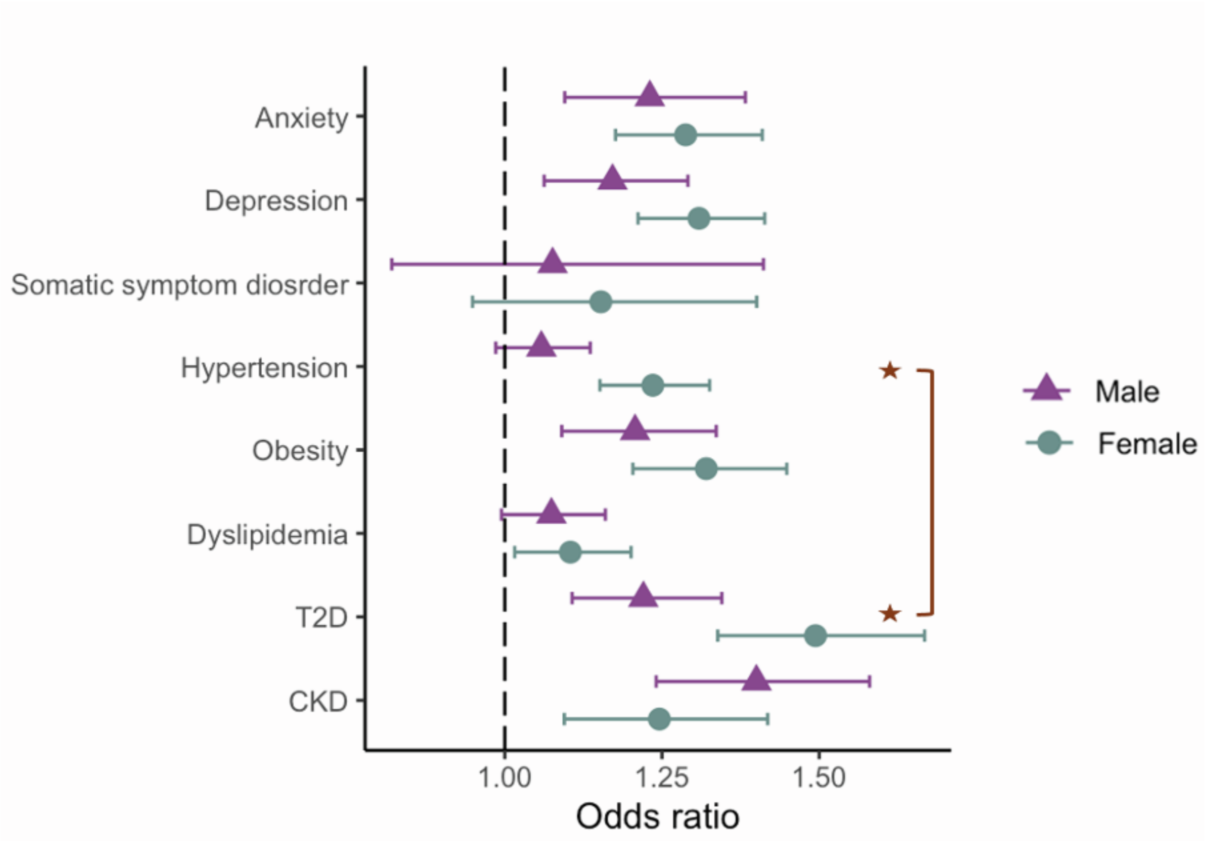

Figure S3. Association of ND-CNVs with internalizing and cardiometabolic conditions for male and female sex. Stars indicate a significant interaction of presence of ND-CNV and sex.

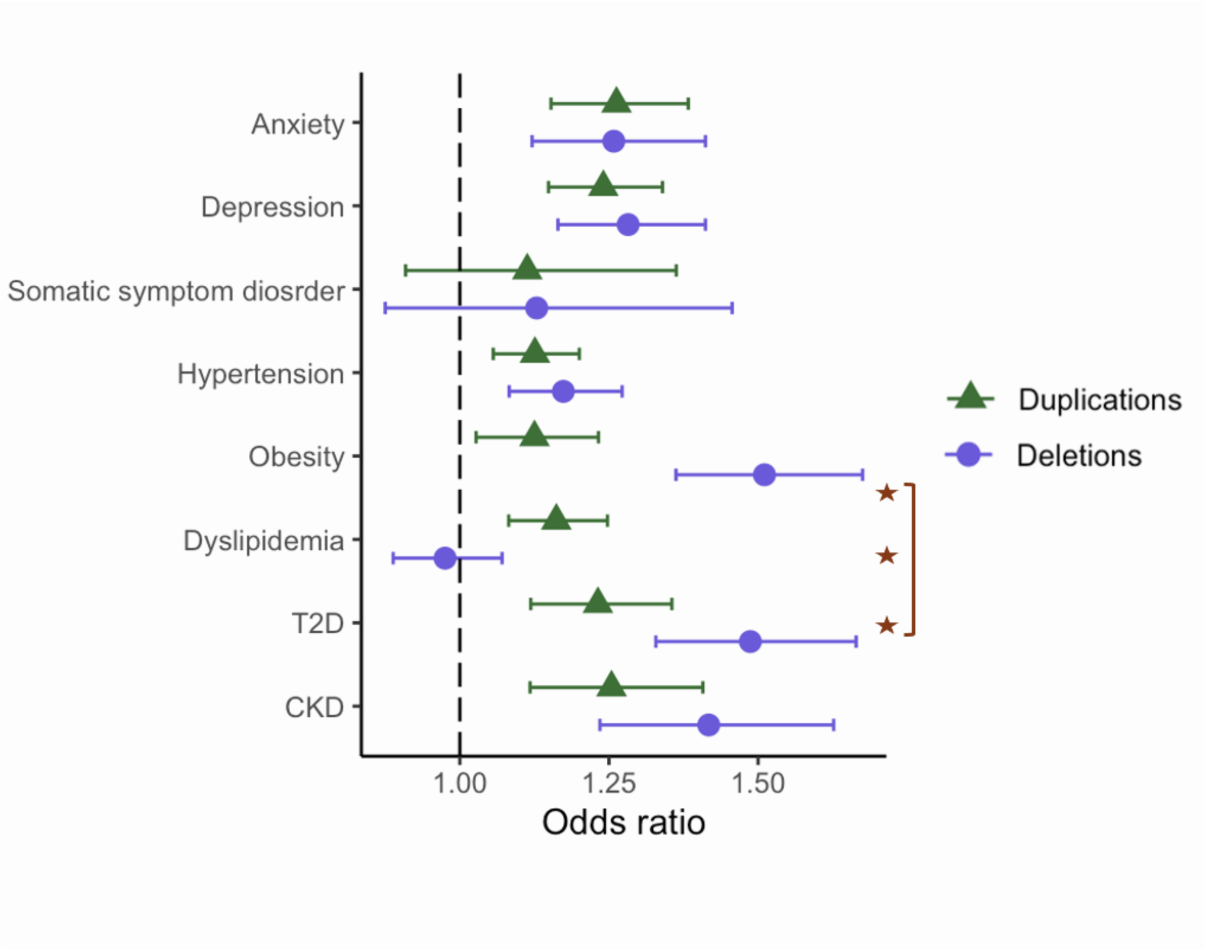

Figure S4. Association of duplications and deletions with internalizing and cardiometabolic conditions. Stars indicate a suggested difference between deletions and duplications (p-value < 0.05).

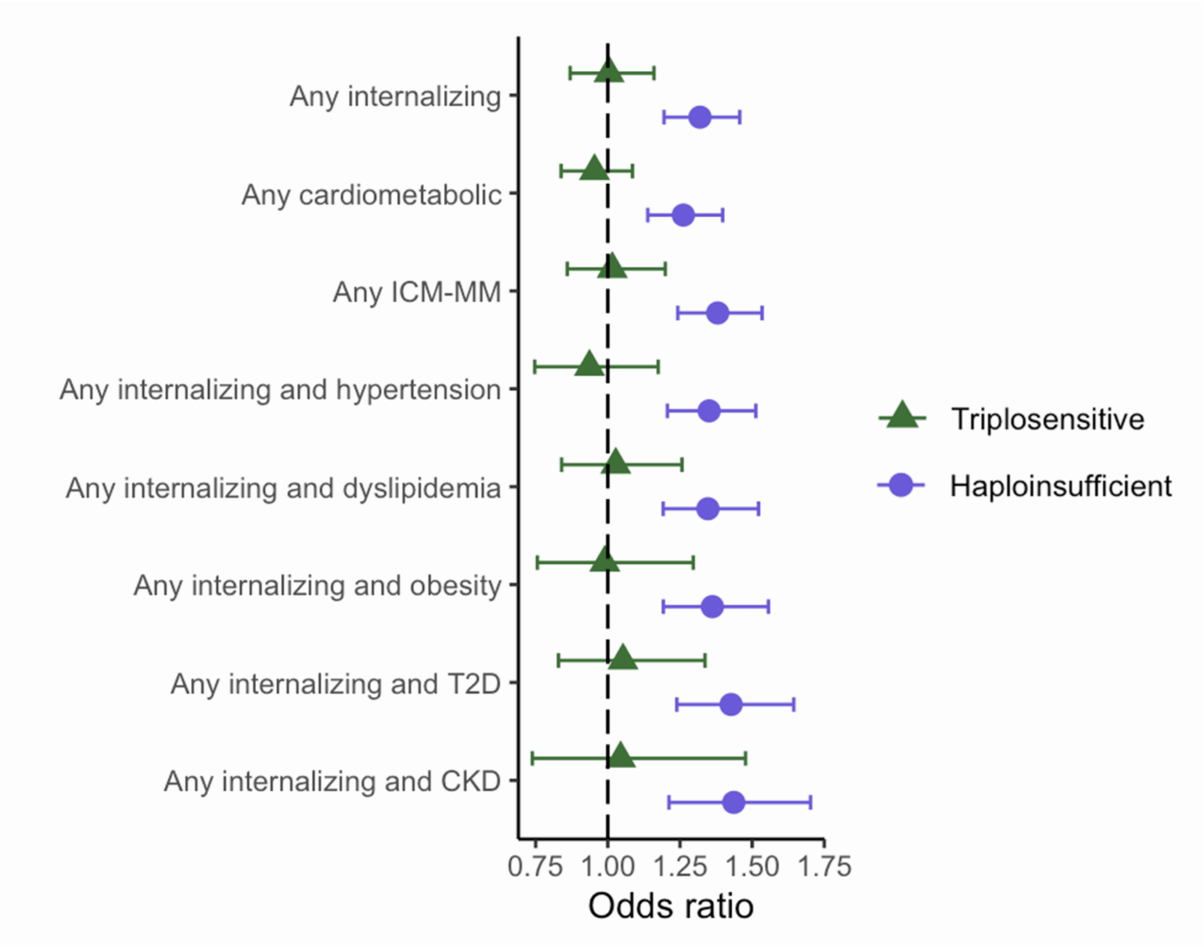

Figure S5. Association of triplosensitive genes in duplications and haploinsufficient genes in deletions with ICM-MM. ORs represent an increase in risk by an increase of one gene.

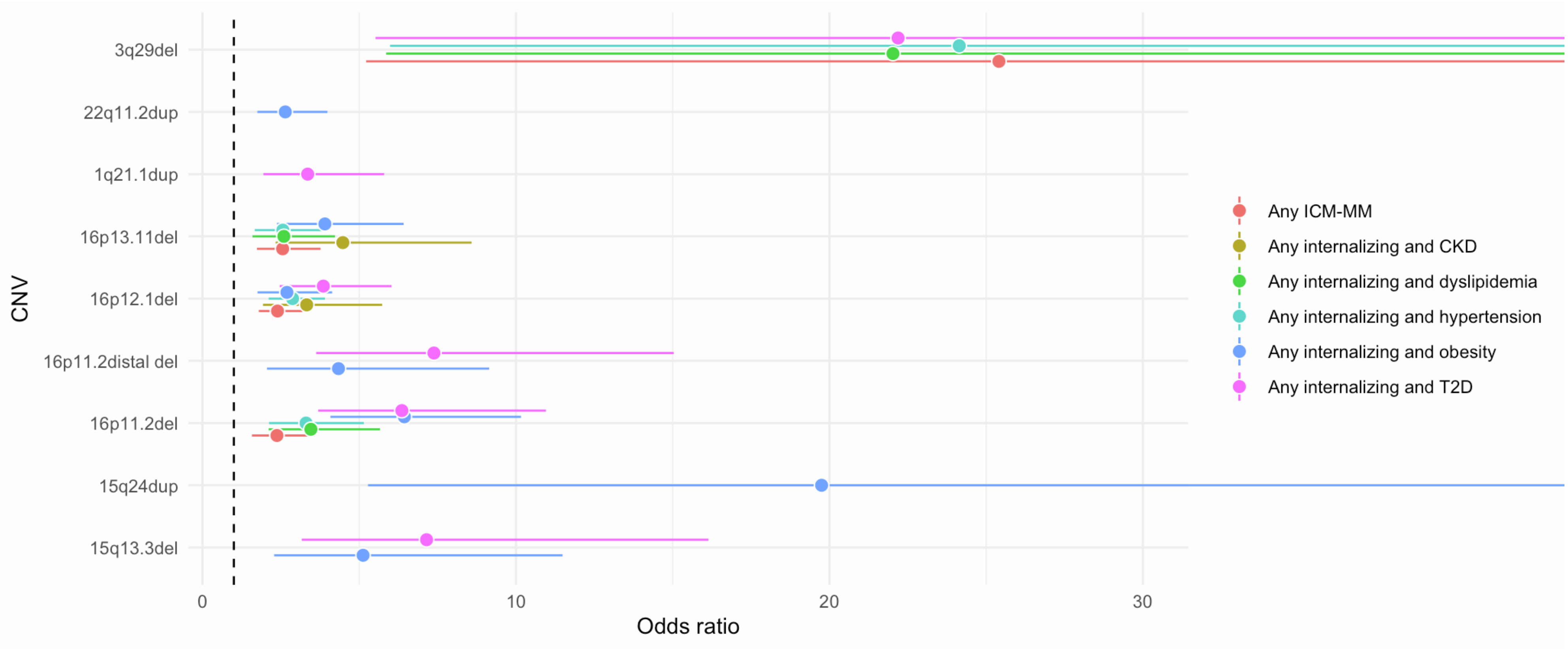

Figure S6. Association of individual ND-CNVs and multimorbidity. Only significant associations after Bonferroni correction for multiple testing are included in the figure.

SUPPLEMENTAL TABLES

Trait	GWAS
MDD	Wray *et al.*, 2018 (UKBB removed) (3)
Anxiety	Meier *et al.*, 2019 (4)
LDL	Willer *et al.*, 2013 (5)
SBP	Keaton *et al.*, 2024 (6) (only ICBP)
BMI	Locke *et al*. 2015 (7)
T2D	Mahajan *et al.*, 2018 (UKBB removed) (8)
CKD	Pattaro *et al.*, 2016 (9)

Table S1. Genome-wide association studies used for polygenic risk score generation.

CNV	Count
Any	7,546
1p36 del	1
TAR del	80
TAR dup	463
1q21.1 del	119
1q21.1 dup	193
NRXN1 del	176
2q11.2 del	34
2q13 del	56
2q13 dup	73
2q37 del	1
3q29 del	9
WH dup	3
WBS del	1
WBS dup	16
8p23.1 del	4
8p23.1 dup	8
EMHT1 dup	1
10q23 del	3
15q11.2 del	1,748
15q11.2 dup	2,284

PWS del	1
PWS dup	19
15q13.3 del	47
15q24 del	1
15q24 dup	9
15q25 del	1
16p13.11 del	140
16p13.11 dup	888
16p12.1 del	260
16p11.2distal del	62
16p11.2distal dup	142
16p11.2 del	123
16p11.2 dup	142
17p13.3 YWHAE del	27
17p13.3 YWHAE dup	8
17p13.3 PAFAH1B1 del	1
17p13.3 PAFAH1B1 dup	3
SMS	2
Potocki Lupski	6
17q11.2 delNF1	10
17q11.2 dup NF1	3
17q12 del	9
17q12 dup	104
22q11.2 del	10
22q11.2 dup	294
22q11.2 distal del	5
22q11.2 distal dup	14

Table S2. Number of individuals with each ND-CNV. ND-CNVs that appeared less than five times are in italics.

	ND-CNV	No ND-CNV
Age at recruitment<br>(Mean (min, max))	56.07 (40,70)	56.55 (37,73)
Townsend deprivation index<br>(Mean (min, max))	-0.86 (-6.26, 10.56)	-1.32 (-6.26, 11.00)
Female<br>(N (%))	3,934 (52.14)	245,589 (54.32)

Table S3. Demographic characteristics of individuals with and without a ND-CNV. NV. Townsend deprivation index is measure of socioeconomic deprivation, with higher value indicating a more deprived environment.

	ND-CNV	(N=7,546)	No ND-CNV	(N=451,937)
	Cases	Frequency (%)	Cases	Frequency (%)
Anxiety	878	11.6	42,365	9.4
Depression	1,335	17.7	65,040	14.4
Somatic symptom disorder	161	2.1	8,513	1.9
Any internalizing disorder	1,685	22.3	84,755	18.8
Hypertension	2,765	36.6	153,829	34.0
Obesity	975	12.9	46,176	10.2
Chronic kidney disease	570	7.6	26,924	6.0
Type II diabetes	870	11.5	39,236	8.7
Dyslipidemia	1,709	22.6	95,960	21.2
Any CM	3,705	49.1	206,240	45.7

Table S4. Counts and frequency for each of the conditions of interest in individuals with and without a ND-CNV.

	OR (CI)	p-value
Anxiety	1.25 (1.16-1.34)	9.16×10^{-10}
Depression	1.24 (1.16-1.31)	5.11×10^{-12}
Somatic symptom disorder	1.13 (0.96-1.32)	0.144
Any internalizing condition	1.22 (1.15 -1.28)	4.50×10^{-12}
Hypertension	1.16 (1.10-1.22)	1.20×10^{-8}
Obesity	1.27 (1.18-1.36)	1.04×10^{-11}
Dyslipidaemia	1.10 (1.22-1.46)	1.69×10^{-3}
Type II diabetes	1.37 (1.27-1.48)	2.43×10^{-17}
Chronic kidney disease	1.33 (1.03-1.16)	1.45×10^{-10}
Any cardiometabolic condition	1.19 (1.13-1.24)	5.37×10^{-12}
Any internalizing and hypertension	1.21 (1.13-1.31)	5.55×10^{-7}
Any internalizing and dyslipidemia	1.27 (1.16-1.38)	1.34×10^{-7}
Any internalizing and obesity	1.48 (1.33-.164)	1.51×10^{-13}
Any internalizing T2D	1.57 (1.38-1.77)	1.66×10^{-6}
Any internalizing and CKD	1.44 (1.24-1.66)	1.09×10^{-6}
Any ICM-MM	1.25 (1.17-1.34)	3.14×10^{-11}

Table S5. Association of individual conditions and ICM-MM phenotypes with the presence of a ND-CNV.

	OR (CI)	p-value
Any ICM-MM	1.18 (1.10-1.26)	1.32×10^{-6}
Any internalizing and hypertension	1.14 (1.05-1.23)	9.56×10^{-4}
Any internalizing and dyslipidemia	1.20 (1.10-1.32)	3.89×10^{-5}
Any internalizing T2D	1.41 (1.24-1.60)	7.11×10^{-8}
Any internalizing and CKD	1.35 (1.17-1.56)	6.53×10^{-5}

Table S6. Association of ICM-MM phenotypes with the presence of a ND-CNV adjusting for BMI. Obesity was not included as an outcome in this analysis.

	OR (CI)	p-value
Any ICM-MM	1.20 (1.11-1.30)	$1.21x10^{-5}$
Any internalizing and hypertension	1.17 (1.07-1.29)	$1.09x10^{-3}$
Any internalizing and dyslipidemia	1.18 (1.06-1.31)	$1.92x10^{-3}$
Any internalizing and obesity	1.48 (1.26-1.61)	$1.30x10^{-8}$
Any internalizing T2D	1.56 (1.35-1.80)	$2.42x10^{-9}$
Any internalizing and CKD	1.37 (1.15-1.63)	$4.20x10^{-4}$

Table S7. Association of ICM-MM phenotypes with the presence of a ND-CNV for individuals with both primary care and HES data (N= 229,951).

	OR (CI)	p-value
Any ICM-MM	1.20 (1.11-1.30)	$2.08x10^{-9}$
Any internalizing and hypertension	1.17(1.07-1.29	$7.64x10^{-6}$
Any internalizing and dyslipidemia	1.18 (1.06-1.31)	$3.84x10^{-6}$
Any internalizing and obesity	1.42 (1.26-1.61)	$8.95x10^{-10}$
Any internalizing T2D	1.56 (1.35-1.80)	$7.06x10^{-10}$
Any internalizing and CKD	1.37 (1.15-1.63)	$4.15x10^{-6}$

Table S8. Association of ICM-MM phenotypes with the presence of a ND-CNV excluding individuals with 16p11.2 proximal and distal deletions (N= 185).

	White		Black		Asian		Mixed		Other	
	N = 432,604		N = 7,170		N = 10,194		N = 2,626		N = 4,095	
	OR (CI)	p	OR (CI)	p	OR (CI)	p	OR (CI)	p	OR (CI)	p
Any ICM-MM	1.25 (1.17-1.34)	$4.20\text{x}10^{-11}$	1.64 (0.80-3.37)	0.171	1.37 (0.65-4.98)	0.191	0.86 (0.26-2.90)	0.815	1.70 (0.17-1.83)	0.330
Any internalizing and hypertension	1.21 (1.12-1.31)	$1.42\text{x}10^{-6}$	1.67 (0.75-3.71)	0.206	1.47 (0.87-2.49)	0.142	0.87 (0.202-3.71)	0.846	0.76 (0.23-2.56)	0.678
Any internalizing and dyslipidemia	1.28 (1.18-1.40)	$3.59\text{x}10^{-8}$	3.99 (0.05-2.91)	0.364	1.03 (0.55-1.94)	0.916	1.31 (0.303-5.71)	0.713	0.30 (0.04-2.19)	0.235
Any internalizing and obesity	1.48 (1.33-1.64)	$4.85\text{x}10^{-13}$	1.78 (0.64-5.01)	0.271	1.14 (0.46-2.84)	0.777	2.47 (0.72-8.48)	0.150	0.02 (0-inf)	0.970
Any internalizing and T2D	1.57 (1.39-1.78)	$1.04\text{x}10^{-12}$	1.42 (0.43-4.60)	0.557	1.82 (1.01-3.29)	0.045	8.71 (0-inf)	0.983	1.05 (0.25-4.29)	0.940
Any internalizing and CKD	1.46 (1.26-1.69)	$5.29\text{x}10^{-7}$	1.61 (0-inf)	0.977	1.80 (0.64-4.98)	0.260	1.75 (0-inf)	0.983	0.01 (0-inf)	0.981

Table S9. Association of ICM-MM phenotypes with the presence of a ND-CNV stratified by ethnicity.

	Male sex	Female sex	Difference
	OR (CI)	OR (CI)	p-value
Any internalizing	1.20 (1.10-1.31)	1.25 (1.16-1.34)	0.512
Any cardiometabolic	1.09 (1.02-1.17)	1.25 (1.17-1.34)	0.004
Any ICM-MM	1.19 (1.08-1.32)	1.31 (1.20-1.43)	0.211
Any internalizing and hypertension	1.12 (0.99-1.26)	1.31 (1.18-1.45)	0.063
Any internalizing and dyslipidemia	1.19 (1.05-1.36)	1.34 (1.19-1.51)	0.214
Any internalizing and obesity	1.39 (1.17-1.66)	1.55 (1.36-1.76)	0.328
Any internalizing and T2D	1.35 (13.1-1.61)	1.78 (1.51-2.10)	0.026
Any internalizing and CKD	1.61 (1.30-1.99)	1.32 (1.08-1.62)	0.181

Table S10. Association of ICM-MM phenotypes with ND-CNV for male and female sex.

	Duplications	Deletions	Difference
	OR (CI)	OR (CI)	p-value
Any internalizing	1.21 (1.13-1.29)	1.26 (1.16-1.38)	0.834
Any cardiometabolic	1.17 (1.10-1.24)	1.17 (1.08-1.26)	0.980
Any ICM-MM	1.21 (1.11-1.32)	1.32 (1.19-1.47)	0.430
Any internalizing and hypertension	1.16 (1.05-1.28)	1.31 (1.17-1.48)	0.281
Any internalizing and dyslipidemia	1.31 (1.18-1.47)	1.20 (1.04-1.38)	0.596
Any internalizing and obesity	1.27 (1.10-1.46)	1.85 (1.59-2.15)	0.002
Any internalizing and T2D	1.45 (1.24-1.70)	1.72 (1.44-2.07)	0.267
Any internalizing and CKD	1.34 (1.11-1.62)	1.61 (1.29-2.02)	0.439

Table S11. Association of ICM-MM phenotypes with duplications and deletions.

	Deletions		Duplications	
	Haploinsufficient genes	Total genes	Triplosensitive genes	Total genes
	OR (CI)	OR (CI)	OR (CI)	OR (CI)
Any internalizing	1.31 (1.19-1.46) *	1.01 (1.00-1.01) *	1.01 (0.86-1.15)	1.01 (1.00-1.01) *
Any cardiometabolic	1.26 (1.14-1.40) *	1.01 (1.00-1.01) *	0.95 (0.84-1.08)	1.01 (1.00-1.01) *
Any ICM-MM	1.38 (1.24-1.53) *	1.01 (1.00-1.01) *	1.01 (0.86 -1.20)	1.01 (1.00-1.01) *
Any internalizing and hypertension	1.35 (1.21-1.51) *	1 (1.00-1.01)	0.94 (0.75-1.17)	1. 01 (1.00-1.01)
Any internalizing and dyslipidemia	1.35 (1.19-1.52) *	1.01 (1.00-1.01) *	1.03 (0.84-1.25)	1.01 (1.00-1.01) *
Any internalizing and obesity	1.36 (1.10-1.55) *	1.01 (1.01-1.03) *	0.99 (0.76-1.30)	1.01 (1.00-1.01) *

Any internalizing and T2D	1.43 (1.24.1.64) *	1.01 (1.01-1.02) *	1.05 (0.83-1.34)	1.01 (1.00-1.01) *
Any internalizing and CKD	1.44 (1.21-1.70) *	1.01 (1.00-1.01)	1.04 (0.74-1.48)	1.01 (1.00-1.01)

Table S12. Association of haploinsufficient and total genes in deletions and triplosensitive and total genes in duplications with ICM-MM. ORs represent odds by an increase of one gene. Asterisks indicate statistical significance after Bonferroni correction for multiple testing.

	ND-CNV*PRS													
	**MDD**		**Anxiety**		**T2D**		**BMI**		**SBP**		**LDL**		**CKD**	
**Outcome**	**OR (CI)**	**p-value**	**OR (CI)**	**p-value**	**OR (CI)**	**p-value**	**OR (CI)**	**p-value**	**OR (CI)**	**p-value**	**OR (CI)**	**p-value**	**OR (CI)**	**p-value**
**Any ICM-MM**	1.01 (0.94, 1.08)	0.857	1.03 (0.96,1.10)	0.438	1.00 (0.93,1.08)	0.983	0.98 (0.91,1.06)	0.668	0.96 (0.89,1.03)	0.269	1.03 (0.96,1.11)	0.351	1.02 (0.95,1.09)	0.606
**Any internalizing and hypertension**	0.96 (0.8, 81.04)	0.323	1.01 (0.93,1.09)	0.842	1.02 (0.94,1.11)	0.616	1.01 (0.92,1.10)	0.902	0.98 (0.90,1.07)	0.678	1.06 (0.97,1.14)	0.183	1.01 (0.93,1.10)	0.775
**Any internalizing and dyslipidemia**	1.04 (0.94, 1.14)	0.468	1.07 (0.97,1.17)	0.176	1.01 (0.92,1.12)	0.795	0.97 (0.87,1.07)	0.500	1.02 (0.93,1.12)	0.651	1.05 (0.95,1.15)	0.332	1.03 (0.94,1.13)	0.535
**Any internalizing and obesity**	1.10 (0.98, 1.23)	0.094	1.15 (1.04,1.28)	0.010	0.97 (0.87,1.09)	0.642	0.98 (0.87,1.10)	0.738	1.02 (0.92,1.15)	0.667	1.02 (0.91,1.13)	0.748	1.07 (0.96,1.19)	0.237
**Any internalizing and T2D**	0.93 (0.82, 1.07)	0.317	1.01 (0.89,1.14)	0.884	1.01 (0.88,1.16)	0.907	0.98 (0.85,1.13)	0.775	1.09 (0.96,1.25)	0.177	1.05 (0.93,1.20)	0.428	0.95 (0.84,1.08)	0.413
**Any internalizing and CKD**	1.06 (0.90, 1.24)	0.486	1.18 (1.01,1.37)	0.037	0.88 (0.75,1.04)	0.143	0.94 (0.79,1.11)	0.470	1.05 (0.90,1.23)	0.549	1.18 (1.01,1.37)	0.039	1.01 (0.87,1.18)	0.893

Table S13. Interaction between presence of ND-CNVs and PRSs on outcome risk. To assess whether the association of common genetic variation and the risk of multimorbidity differs in individuals with and without a ND-CNV, logistic regression analyses were performed for each of the outcomes, including the main effects of each ancestry-adjusted PRS, ND-CNV and the interaction term PRS*ND-CNV. The odds ratios and p-values correspond to the ND-CNV*PRS interaction term for each of the seven PRSs examined.

PRS	OR (CI)	p-value
MDD	1.05 (1.02-1.08)	1.21×10^{-4}
Anxiety	1.06 (1.03-1.08)	2.21×10^{-6}
T2D	1.01 (0.99-1.04)	0.296
BMI	1.02 (1.00-1.05)	0.087
SBP	1.01 (0.99-1.04)	0.344
LDL	1.00 (0.97-1.02)	0.980
CKD	1.04 (1.01-1.06)	2.52×10^{-3}

Table S14. Association of ND-CNV with each of the PRSs.

SUPPLEMENTAL REFERENCES

1. Eto F, Samuel M, Finer S. MULTIPLY-Initiative: Version 1.1 [Internet]. Zenodo; 2023 [cited 2023 Aug 31]. Available from: https://zenodo.org/record/7643566

2. Baksh RA, Pape SE, Chan LF, Aslam AA, Gulliford MC, Strydom A. Multiple morbidity across the lifespan in people with Down syndrome or intellectual disabilities: a population-based cohort study using electronic health records. Lancet Public Health. 2023 Jun 1;8(6):e453–62.

3. Wray NR, Ripke S, Mattheisen M, Trzaskowski M, Byrne EM, Abdellaoui A, et al. Genome-wide association analyses identify 44 risk variants and refine the genetic architecture of major depression. Nat Genet. 2018 May;50(5):668–81.

4. Meier SM, Trontti K, Purves KL, Als TD, Grove J, Laine M, et al. Genetic Variants Associated With Anxiety and Stress-Related Disorders: A Genome-Wide Association Study and Mouse-Model Study. JAMA Psychiatry. 2019 Sep 1;76(9):924–32.

5. Willer CJ, Schmidt EM, Sengupta S, Peloso GM, Gustafsson S, Kanoni S, et al. Discovery and Refinement of Loci Associated with Lipid Levels. Nat Genet. 2013 Nov;45(11):1274.

6. Keaton JM, Kamali Z, Xie T, Vaez A, Williams A, Goleva SB, et al. Genome-wide analysis in over 1 million individuals of European ancestry yields improved polygenic risk scores for blood pressure traits. Nat Genet. 2024 May;56(5):778–91.

7. Locke AE, Kahali B, Berndt SI, Justice AE, Pers TH, Day FR, et al. Genetic studies of body mass index yield new insights for obesity biology. Nature. 2015 Feb;518(7538):197–206.

9. Mahajan A, Taliun D, Thurner M, et al. Fine-mapping type 2 diabetes loci to single-variant resolution using high-density imputation and islet-specific epigenome maps. Nat Genet. 2018;50(11):1505-1513. doi:10.1038/s41588-018-0241-6

10. Pattaro, C., Teumer, A., Gorski, M. et al. Genetic associations at 53 loci highlight cell types and biological pathways relevant for kidney function. Nat Commun 7, 10023 (2016). https://doi.org/10.1038/ncomms10023
